# Supplementary material for: Crumbs2 acts as a dynamic cellular sensor triggering stable podocyte cell-matrix anchorage
Source: Biophys J. 2025 Jul 22;125(12):2945–63. doi: 10.1016/j.bpj.2025.07.020 (PMC13507169; doi:10.1016/j.bpj.2025.07.020)
Supplement: Supplementary file 1 [file mmc1.pdf]

Biophysical Journal, Volume *125*

Supplemental information

Crumbs2 acts as a dynamic cellular sensor triggering stable podocyte cell-matrix anchorage

Rohan Bhatia, Annika Möller-Kerutt, Sven Gerlach, Yannick Herfs, Rebecca Rixen, Hermann Pavenstädt, Bernd Hoffmann, Rudolf Merkel, Ulrich Kubitscheck, Thomas Weide, and Jan Peter Siebrasse

Biophysical Journal

Supplemental Materials

This PDF file includes:

Figs. S1 to S11

Other Supplemental Materials for this manuscript include the following:

Movies S1 to S5

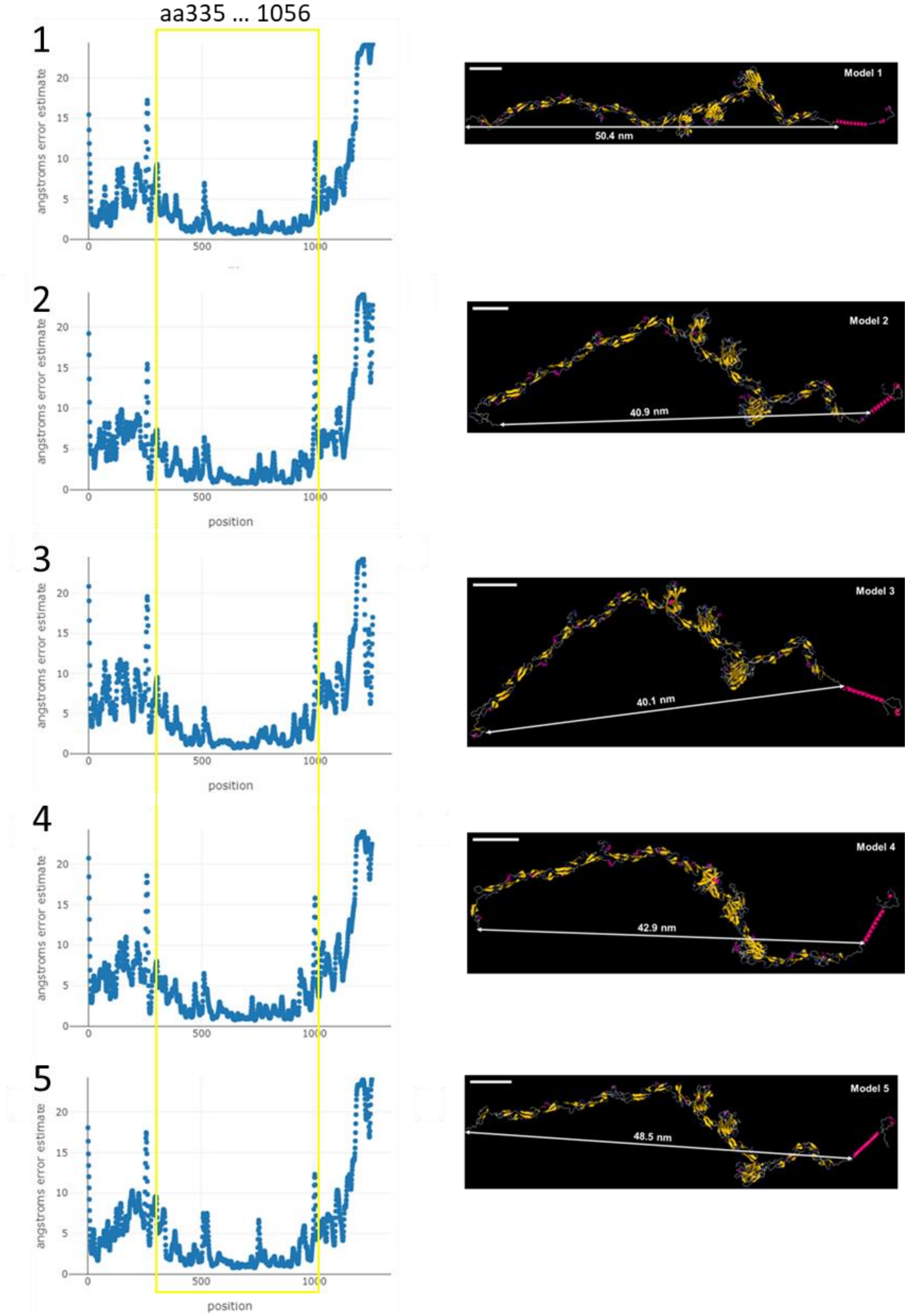

Fig. S1: In-silico prediction of the CRB2 protein structure.
Five different computational models of CRB2 wt computed with the RosettaFold software [23] (right column) with their respective error estimate (left column).

Western blots

Western blots were prepared to compare the expression level of constitutively or inducibly controlled GFP tagged variants of CRBs proteins against α-tubulin which was chosen as the housekeeping gene. All CRB variants showed typical double bands at the expected sizes (CRB2 wt, CRB2ΔFBM and CRB2ΔPBM: ~180-200 kDa.; CRB2ΔECD : ~70 kDa.; CRB3A wt: ~55 kDa. and CRB3ΔICD: < 55 kDa.) indicating proper glycosylation and transfer to the PM of these CRB proteins in all cell lines as shown in Figure 2 and Figure S11. An additional, non-specific band at approximately 130 kDa. was also visible, likely due to antibody cross-reactivity as it appeared also for the control cell line (represented as * in Figure S2).
Comparing the expression level for the constitutively expressing cells (Figure S2A), CRB 3A and 3ΔICD were clearly similarly expressed as the housekeeping gene whereas CRB2 was not. On the other hand, in the case of the inducible promoter (Figure S2B), all CRBs variants showed lower expression as compared to the housekeeping gene. Weaker α-tubulin bands were visualized for the constitutive CRB3ΔICD and inducible CRB2ΔECD owing to lower cell counts at harvest.

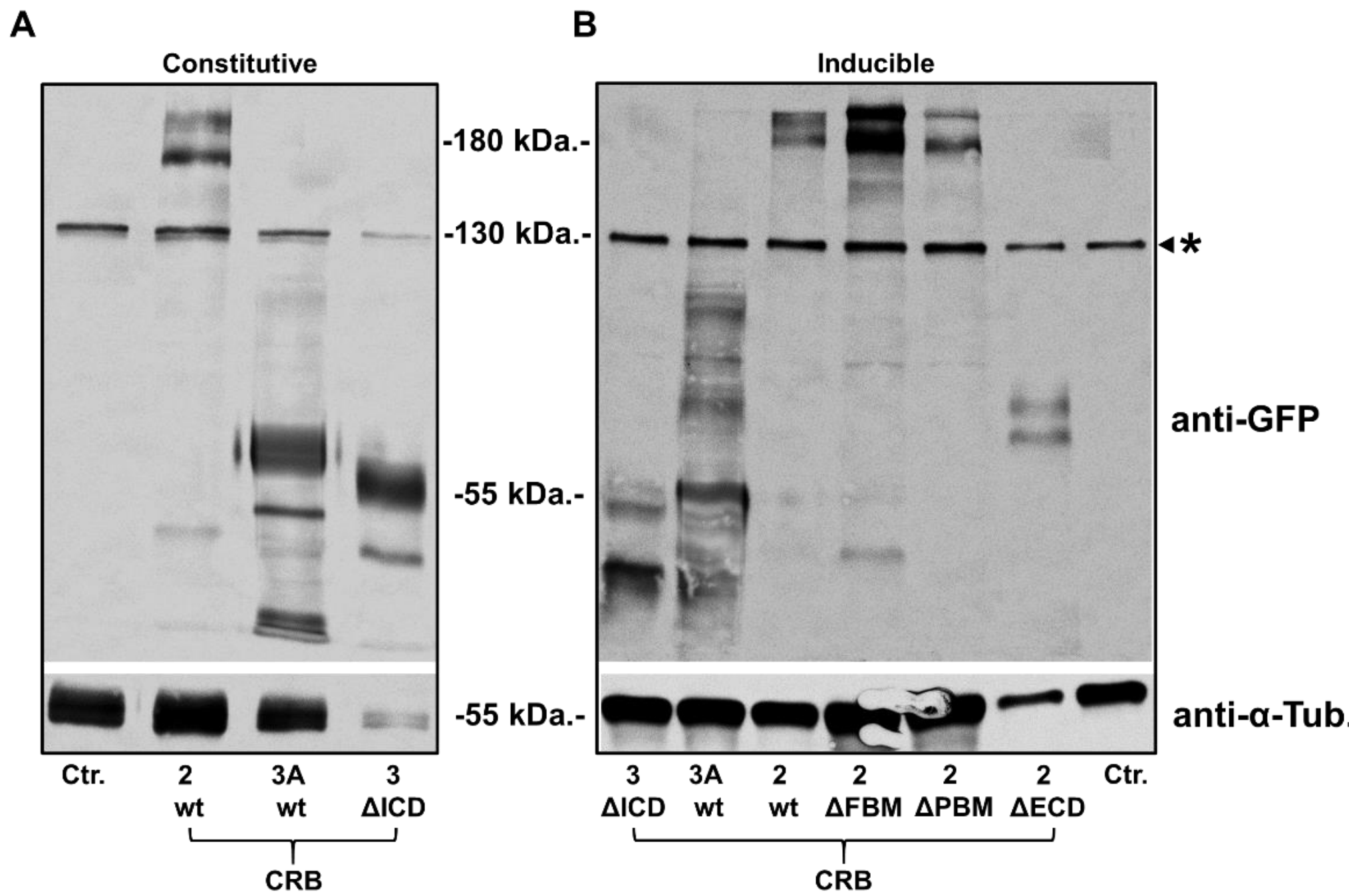

Figure S2: Western Blot analysis of AB8 cells.
(A) Constitutively expressing GFP tagged versions of CRB3ΔICD, CRB3A wt and CRB2 wt along with a cell line not expressing any GFP tagged protein (Ctr.). (B) Inducible GFP tagged versions of CRB3ΔICD, CRB3A wt, CRB2 wt, CRB2ΔFBM, CRB2ΔPBM and CRB2ΔECD along with a cell line not expressing any GFP tagged protein (Ctr.). Induction was initiated by treatment with 200 mM doxycycline for 48 hours. All tested CRB variants show the characteristic double band at the expected sizes. CRB2 wt, CRB2ΔFBM and CRB2ΔPBM: ~180-200 kDa.; CRB2ΔECD: ~70 kDa.; CRB3A wt: ~55 kDa. and CRB3ΔICD: < 55 kDa., which suggests proper glycosylation and folding of the protein. (*) represents the observed cross activity from the GFP antibody at ~130 kDa.

FRAP_measurements

For FRAP measurements three different region of interest (ROI) with diameters of 2 µm were defined. ROI1 served as bleach mask, ROI2 was used to detect bleaching during image recoding and ROI3 provided the background signal (see Fig. S2A and C). In ROI1, GFP fluorescence was bleached by 5 repeated scans with 100% transmission of the argon (488nm and 514nm) and UV lasers (405nm) (Fig. S2B and D). When the bleach ROI was placed in the brighter contact region of two adjacent cells like in Fig. S2A the recording was categorized as “cell-cell membrane interface” and when the bleach ROI was located somewhere within the cell away from the cell-cell contacts like in Fig. S2C it was categorized as “free plasma membrane”. For the free plasma membrane recordings we avoided the ER and the direct nuclear region or any visible intracellular vesicles (Fig. S2 C). Five pre-bleach images were taken and the fluorescence recovery was then recorded for 120 s after bleaching. Raw data for a single measurement were shown in Fig. S2E. The bleach and background corrected curves of 90 measurements were then averaged (Fig. S2F). However, time constants and maximum recovery was calculated from single curves (Fig. S2F; blue triangles).

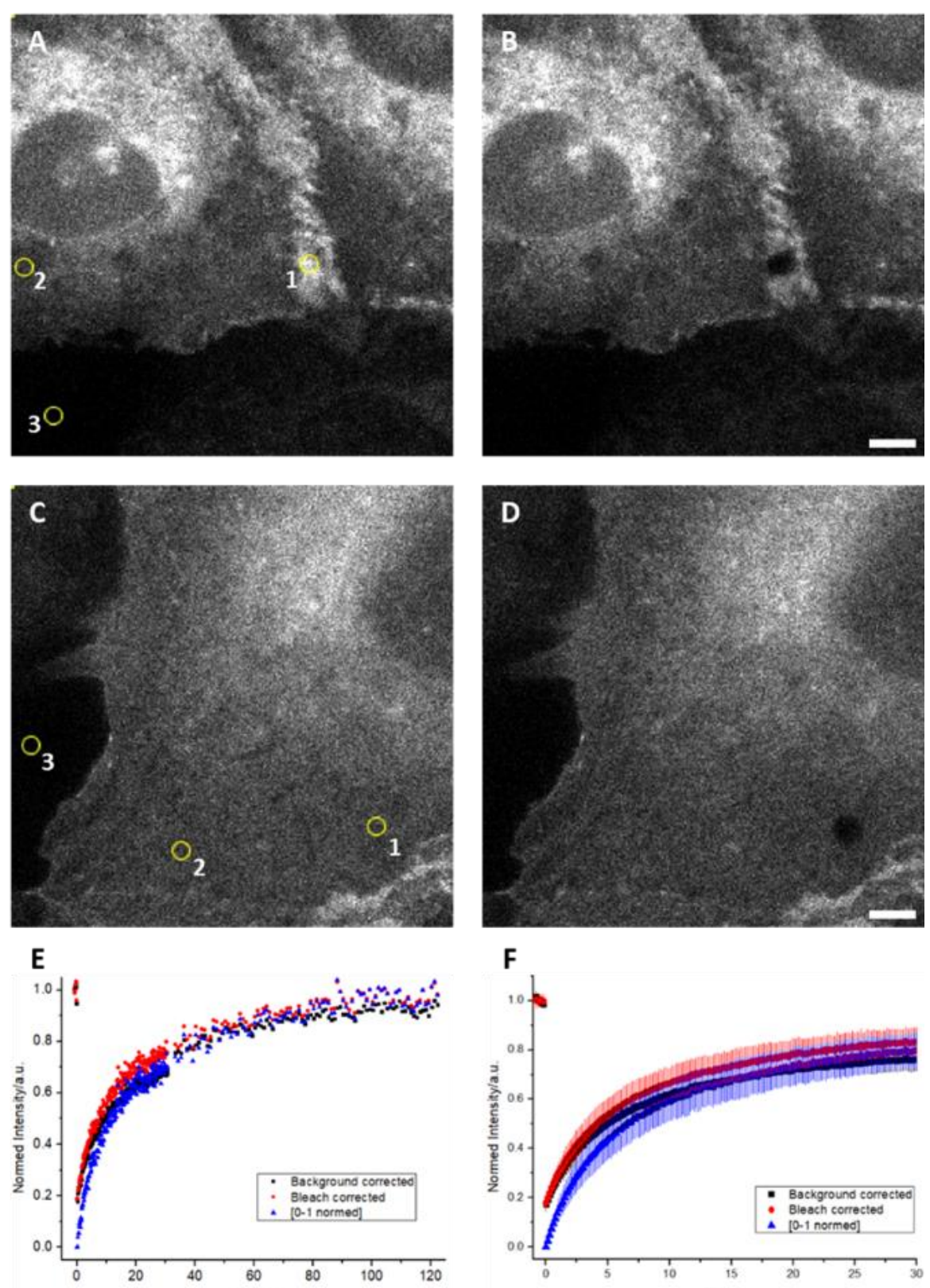

Fig. S3: FRAP Measurements of AB8/13 podocytes expressing CRB2 wt. Scale bar 5 µm
(A) Placement of the three ROI’s for measuring the mobility at the membrane-membrane contact interface. Position 1 yielded the signal of the ROI in question, $F_1(t)$. To monitor the bleaching during recording a second ROI was placed somewhere else in the membrane yielding $F_2(t)$. For background correction we used a third ROI outside the cell (3) yielding F_3. From the three intensity values we calculated a normalized recovery signal as a function of time according to:

$$f(t) = \frac{(F_1(t) - F_3) \cdot (F_2(0) - F_3)}{(F_1(0) - F_3) \cdot (F_2(t) - F_3)}$$

Equation 1

$F_1(0)$ and *$F_2(0)$* correspond to the prebleach intensity values. (B) First image after bleaching the cell shown in (A). (C) Placement of the three ROIs for measuring the mobility in the free plasma membrane. (D) First image after bleaching the cell shown in (C). (E) Raw data for a single FRAP measurement. (F) Averaged data curve from 90 measurements recorded from three independent experiments. The vertical lines display the standard deviation of the data at the individual time points. To illustrate the rapid recovery, only the first 30 seconds of the acquired data are shown. Both the individual FRAP curves and the averaged curves were parameterized by the following double-exponential growth curve:

$$f(t) = A_1 \cdot (1 - e^{-t/\tau_1}) + A_2 \cdot (1 - e^{-t/\tau_2})$$

Equation 2

The time constant τ_1 was interpreted as a measure of the diffusion speed, and the amplitude A_1 as the mobile fraction. We found that A_2 was generally small (about 5% of A_1) and τ_2 was very large (about $10\tau_1$). This term was more dependent on details of bleaching during the monitoring phase and did not reflect the true mobility behavior of the proteins.

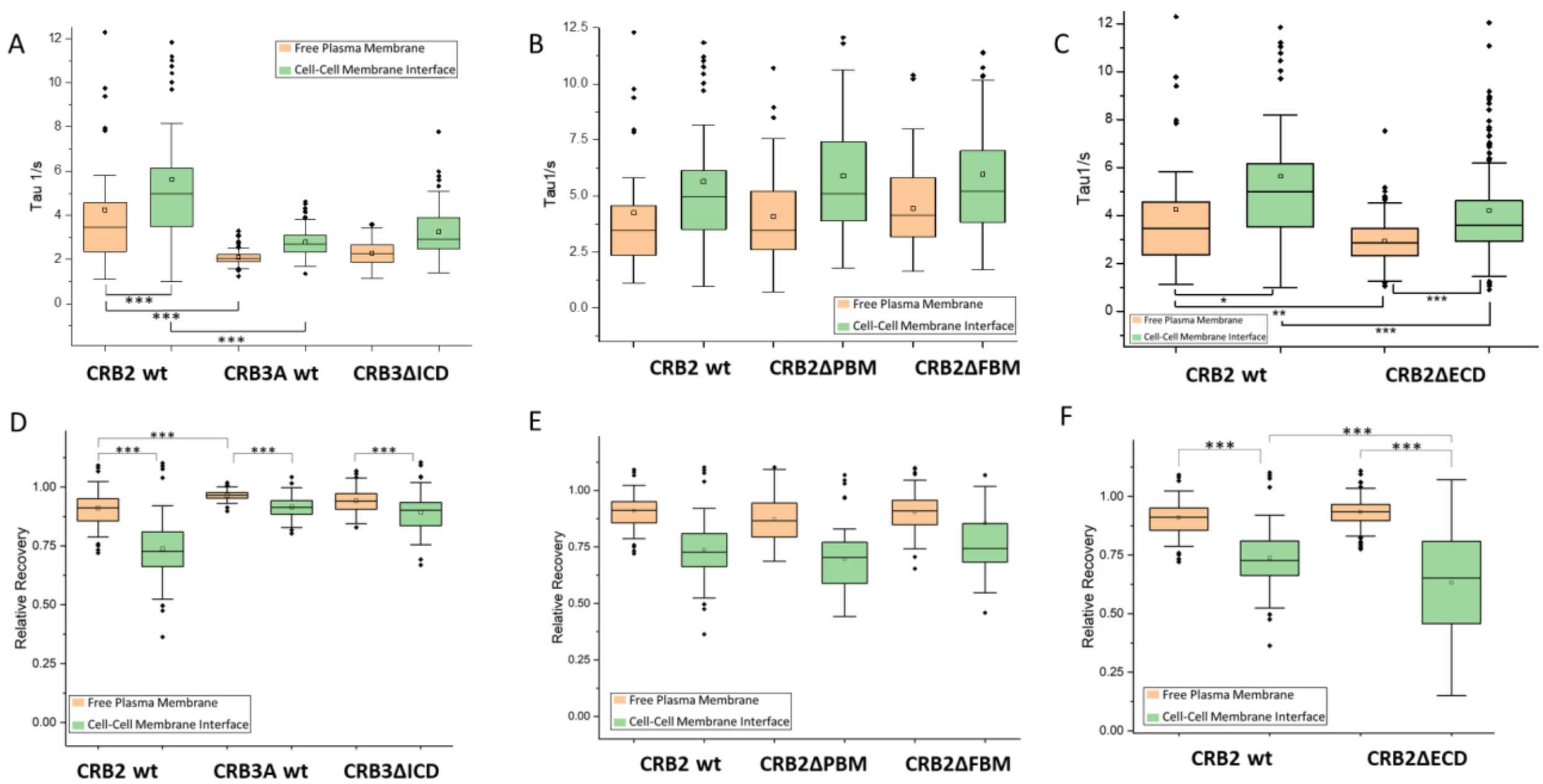

Fig. S4: Statistical analysis of FRAP measurements.

Each individual measurement was fitted to a bi-exponential curve as described. All single τ1 and maximum recovery values were plotted as box plots and tested for significance (*** - p<0.001, ** - p<0.005, * - p<0.01,). (A) CRB2 wt, CRB3A wt and CRB3ΔICD in free plasma membrane. (B) CRB2 wt, CRB2ΔPBM and CRB2ΔFBM in free plasma membrane regions. (C) CRB2 wt and CRB2ΔECD in free plasma membrane regions. (D) CRB2 wt, CRB3A wt and CRB3ΔICD at the cell-cell membrane interface. (E) CRB2 wt, CRB2ΔPBM and CRB2ΔFBM at the cell-cell membrane interface. (F) CRB2 wt and CRB2ΔECD at the cell-cell membrane interface.

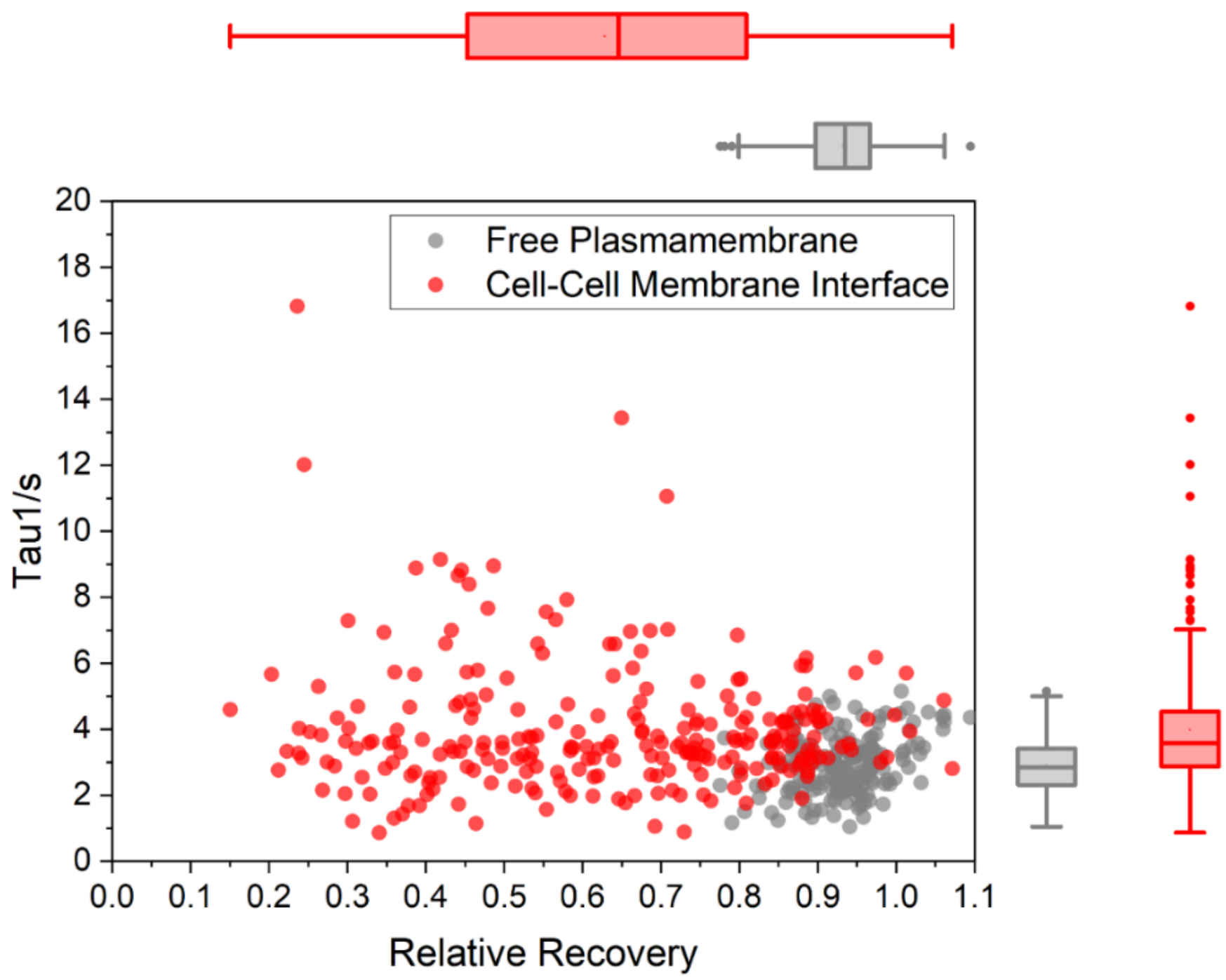

Fig. S5: Single FRAP Measurements of CRB2ΔECD in AB8/13 podocytes.

Combined scatter and box plot of all time constants τ_1 versus the respective maximum relative recovery values A_1 from single bi-exponential fits of all CRB2ΔECD FRAP measurements. For this mutant two complete triplicate sets of experiments comprising 30 individual measurements each corresponding to a total number of 180 single measurements were conducted.

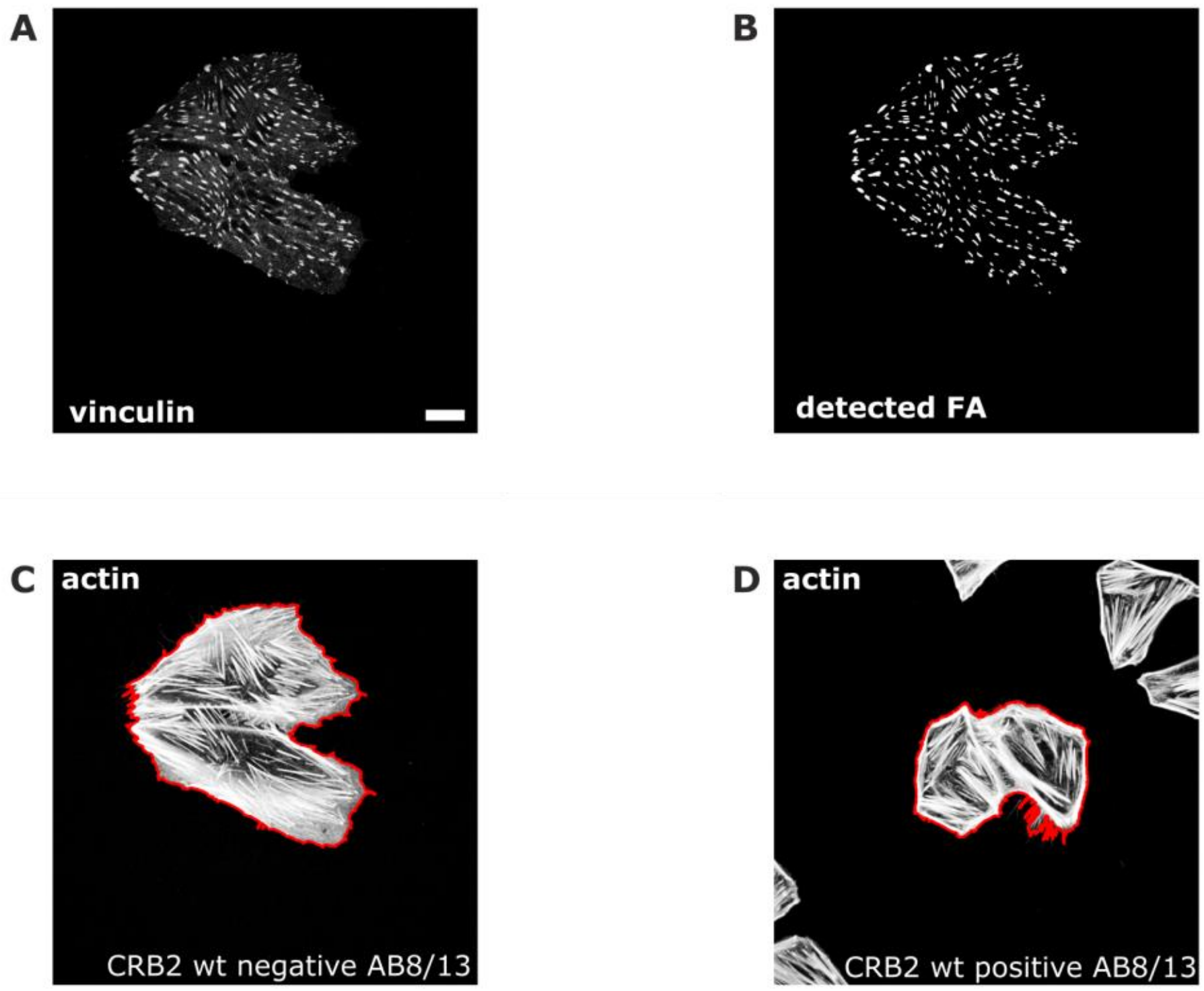

Fig. S6: Quantification of focal adhesions and actin distribution in AB8/13 cells.

(A) Immunofluorescent image of vinculin in AB8/13 cells. (B) Binary mask generated from (A) by z-scoring. (C) Automated detection of actin signal in CRB2 wt negative cell duplets. (D) Automated detection of actin signal in CRB2 wt positive cell duplets. In C and D the cell mask is indicated by a red line. Scale bar, 20 µm, applies to all.

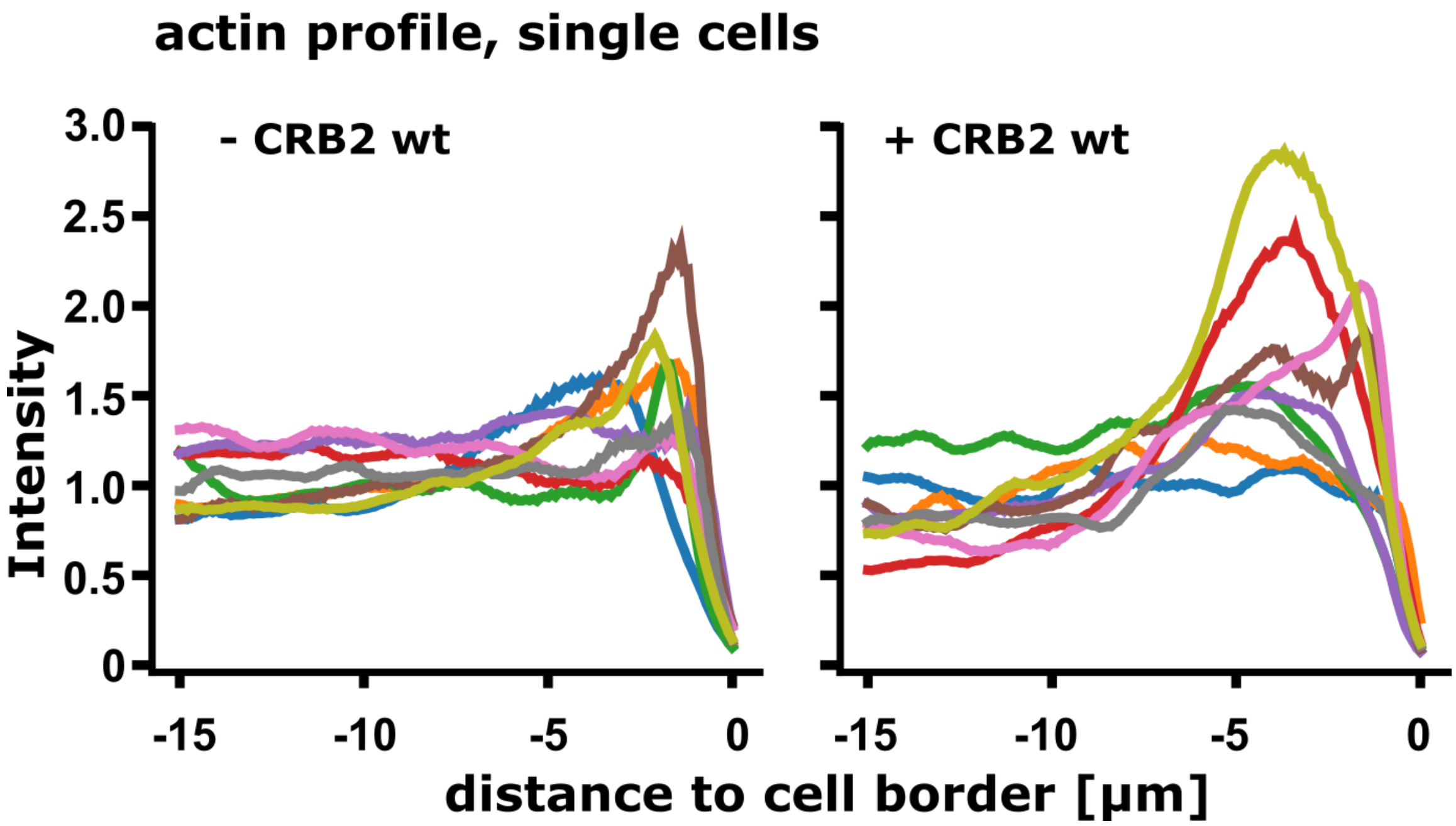

Fig. S7: Distribution of filamentous actin in nine isolated AB8/13 podocytes. Plotted are the mean grey value for each ring normalized to the mean grey value of the entire cell area. Left: CRB2-negative cells, right: CRB2-positive cells.

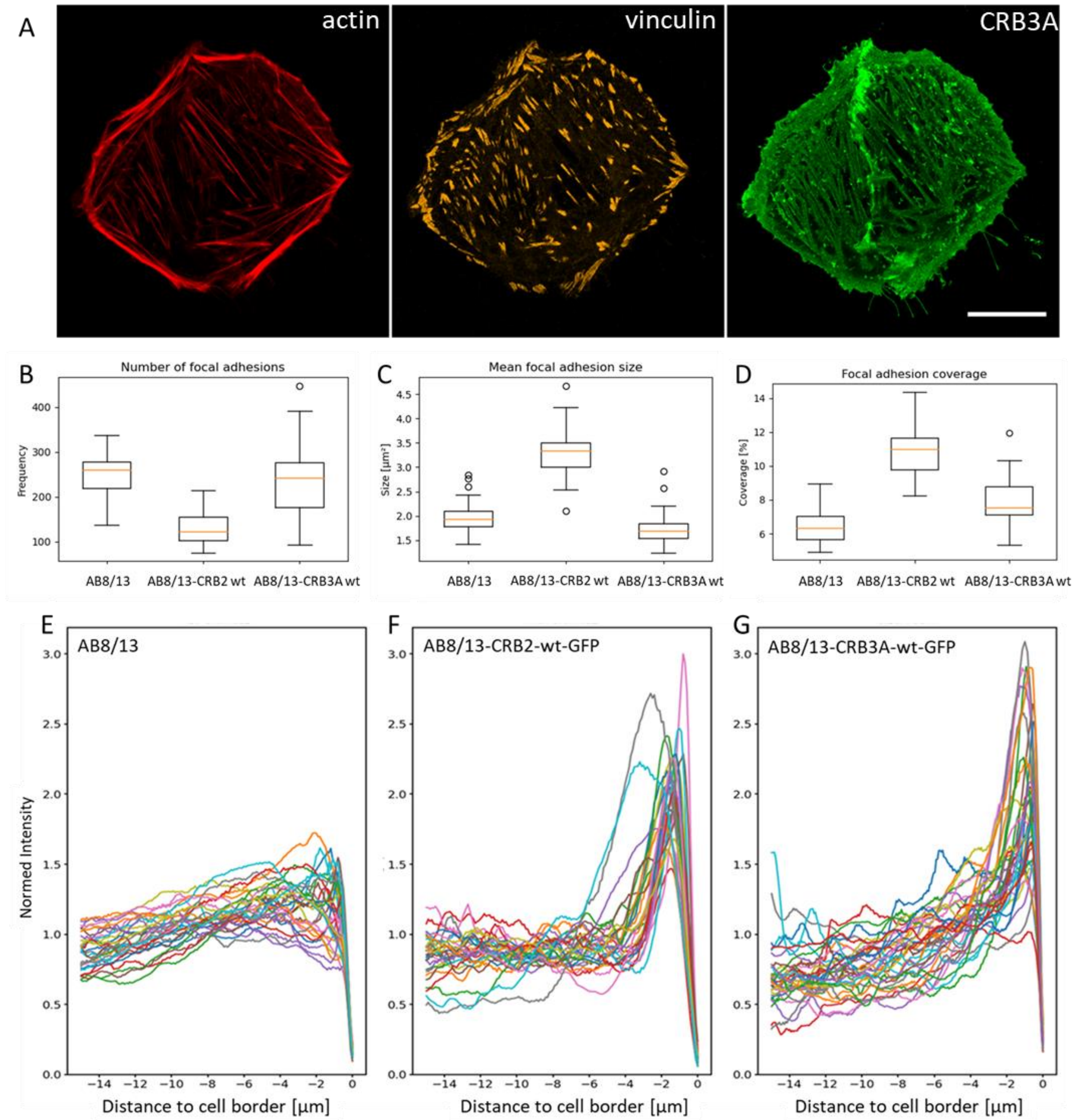

Fig. S8: Quantification of focal adhesions and actin distribution in CRB2-GFP and CRB3A-GFP AB8/13 cell duplets. (A) Immunofluorescence image of actin, vinculin and CRB3A-wt-GFP in AB8/13 cell duplets (scale bar, 20µm, applies to all). (B) Number of focal adhesion in AB8/13, AB8/13-CRB2-wt-GFP and AB8/13-CRB3A-wt-GFP. (C) Mean focal adhesion size in AB8/13, AB8/13-CRB2-wt-GFP and AB8/13-CRB3A-wt-GFP. (D) Focal adhesion coverage in AB8/13, AB8/13-CRB2-wt-GFP and AB8/13-CRB3A-wt-GFP. In B through D dots are results from individual duplets, horizontal bars depict ensemble quartiles and median. (E) Actin intensity profiles in AB8/13. (F) Actin intensity profiles in AB8/13-CRB2-wt-GFP. (G) Actin intensity profiles in AB8/13-CRB3A-wt-GFP.

Wound healing assay

The influence of CRB2 wt overexpression on cell migration was addressed using wound healing assays. Non-induced and doxycycline induced AB8/13 CRB2 wt cells were imaged over 24 h after applying a scratch through the confluent cell layer. Wound areas were recorded by light transmission microscopy at 0h and 24 h (Fig. S7A). Each condition was tested six times. Uninduced AB8/13 cells (-Dox) show a smaller open wound area than podocytes expressing CRB2 wt after 24 h (Fig. S7B).

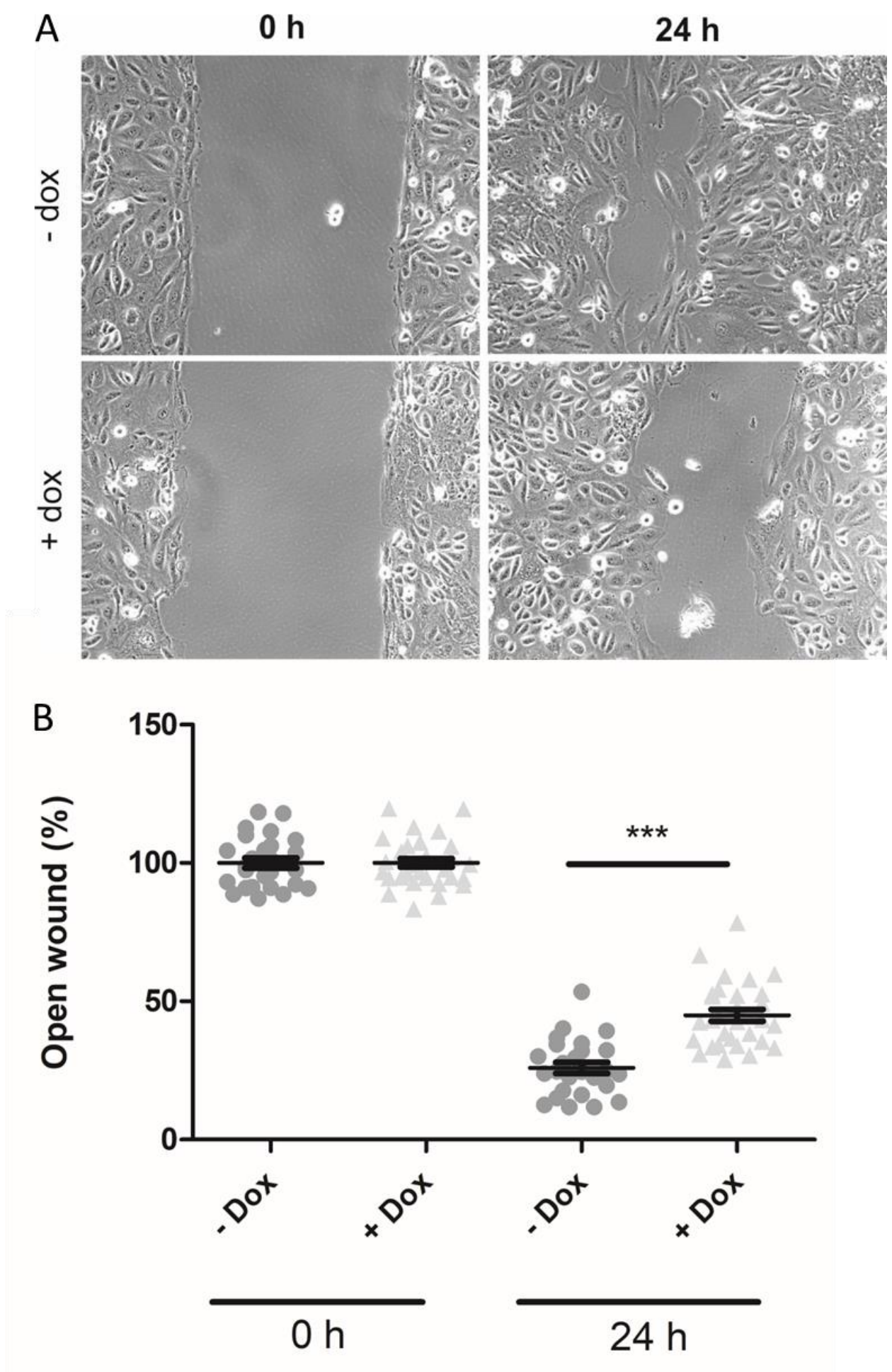

Fig. S9: Analysis of migratory behavior of CRB2 wt cells via wound healing assays.

(A) Migration of AB8/13 podocytes in which the expression of CRB2 wt was not induced (- dox) or was induced (+ dox) with doxycycline. The cell monolayer was imaged immediately after scratching and 24 hours later. (B) Percentage of cell coverage in the beginning and after 24 h of cell migration (*** $p \leq 0.001$).

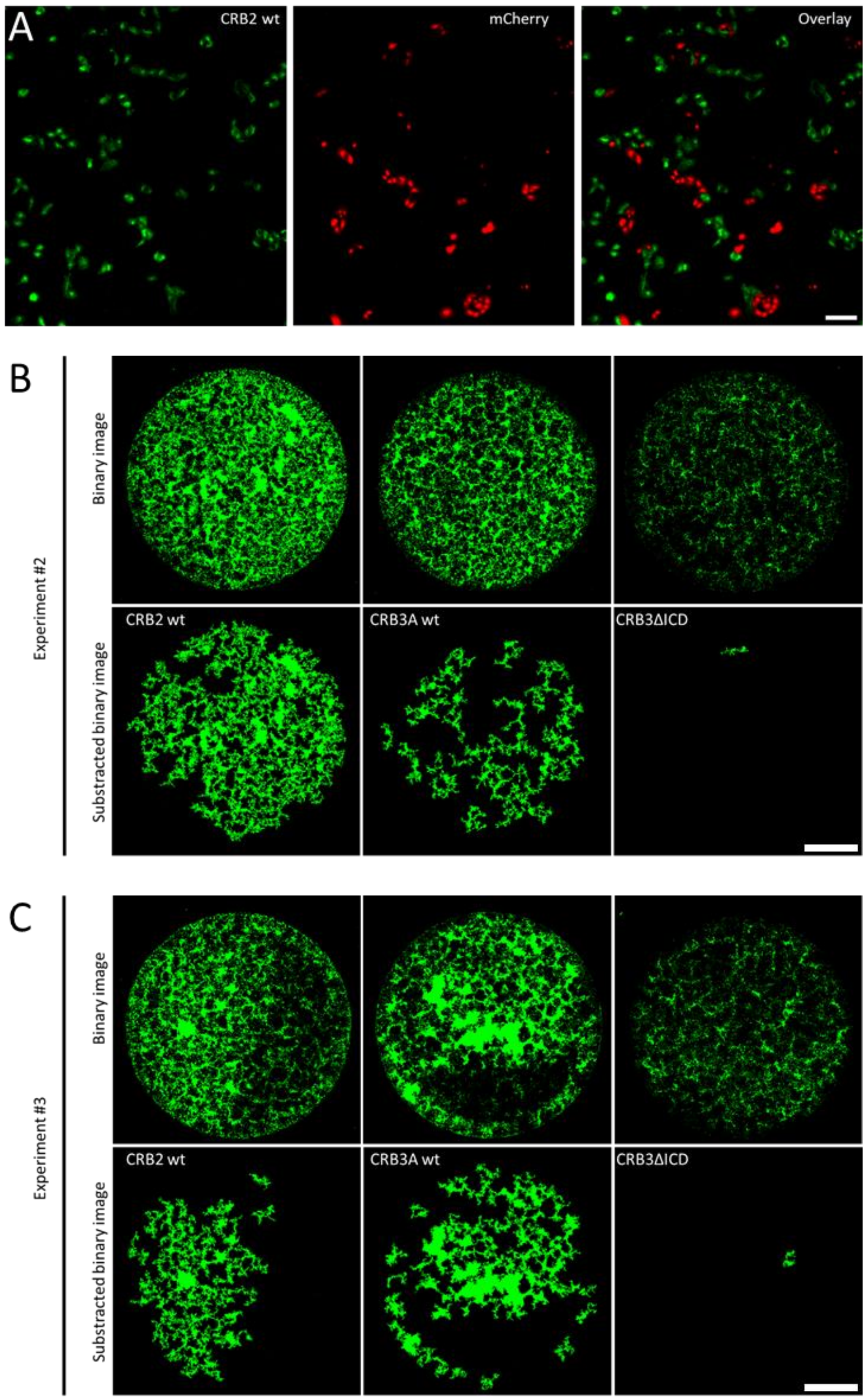

Fig. S10: Growth pattern analysis.

Co-cultivation of AB8/13 podocytes expressing either CRB2-wt-GFP or only mCherry. The complete cell culture dish was imaged using a confocal laser microscope (10x air objective, NA 0.45). (A) Cell distribution after one day of co-culture (size bar, 200 µm). (B) and (C) show the binary and subtracted binary images of two additional independent experiments after seven days (size bar, 5 mm).

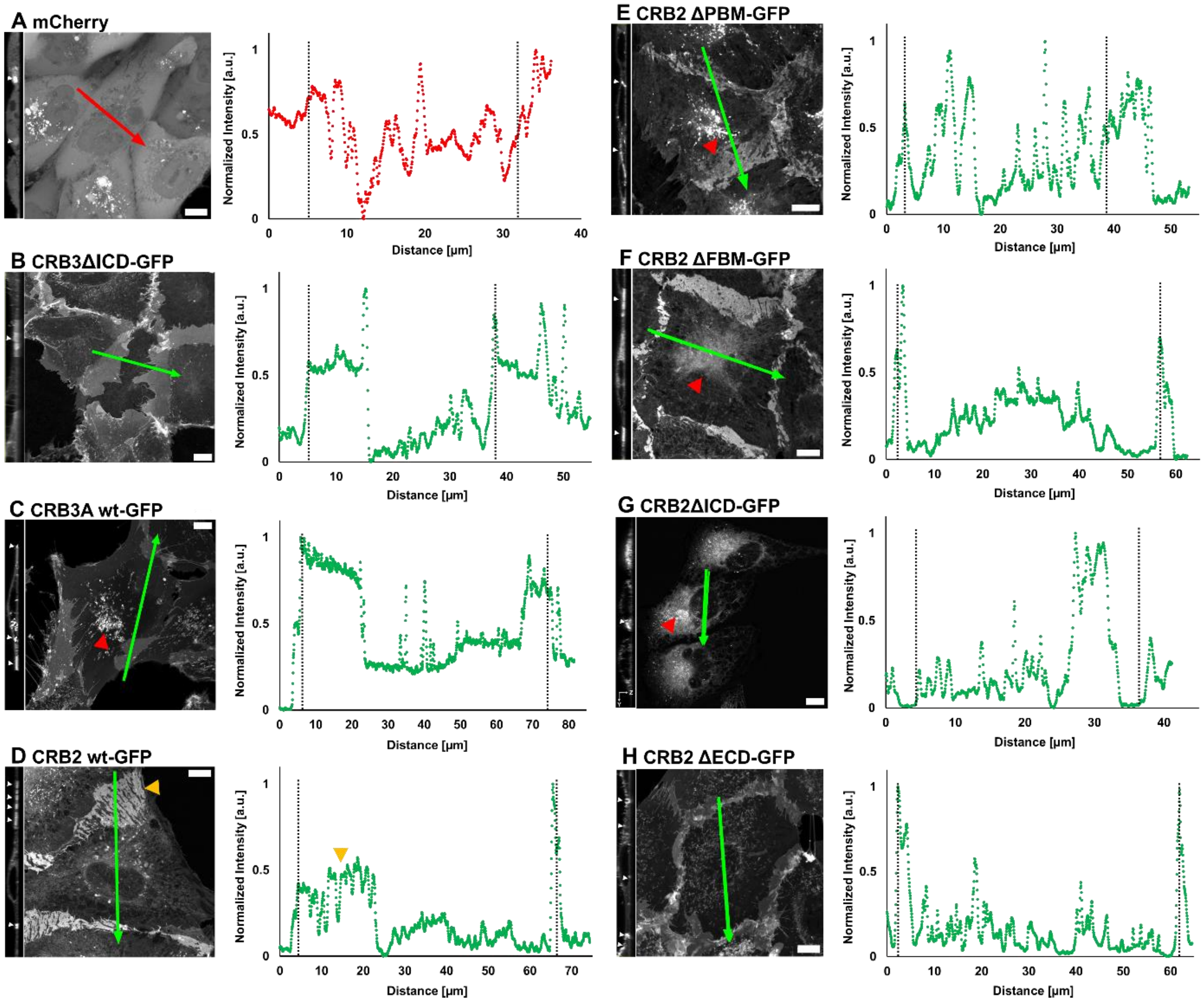

Fig. S11: Sub-cellular protein localization within CRBs isoforms.

(A) mCherry, (B) CRB3ΔICD-GFP, (C) CRB3A wt-GFP; (D) CRB2 wt-GFP, (E) CRB2 ΔPBM-GFP, (F) CRB2 ΔFBM-GFP, (G) CRB2ΔICD-GFP, and (H) CRB2ΔECD-GFP cell lines. Maximum intensity projections of representative Z-stacks (left) along with line profiles (right). Red arrowheads indicate ER localization, yellow arrowheads indicate a CRB2 wt specific morphology. Right figures: distance versus normalized intensity plot shown for the drawn line profile. Dotted lines represent cellular edges, where two cell membranes overlap. All images were acquired with a 40X water immersion objective; NA 1.2 on Zeiss LSM 880 with Airyscan detector. Scale bar: 10 µm.

It is obvious that the CRB2 mutants that we used in the FRAP experiments also show enrichment in the cell-cell membrane interfaces, but intriguingly only CRB2 wt shows a specific distribution within these cell-cell contact regions. Instead of a relative constant intensity we here see dark finger-like structures devoid of the CRB2 wt-GFP. From the immunostaining shown in Fig. 6 we can suppose that the dark regions correspond to focal adhesions according to the vinculin staining.
