# Supplementary material for: Crumbs2 acts as a dynamic cellular sensor triggering stable podocyte cell-matrix anchorage
Source: Biophys J. 2025 Jul 22;125(12):2945–63. doi: 10.1016/j.bpj.2025.07.020 (PMC13507169; doi:10.1016/j.bpj.2025.07.020)
Supplement: Supplementary file 2 [file mmc8.pdf]

Biophysical Journal
Article

Crumbs2 acts as a dynamic cellular sensor triggering stable podocyte cell-matrix anchorage

Rohan Bhatia,[1] Annika Möller-Kerutt,[2] Sven Gerlach,[3] Yannick Herfs,[3] Rebecca Rixen,[2] Hermann Pavenstädt,[2] Bernd Hoffmann,[3] Rudolf Merkel,[3] Ulrich Kubitscheck,[1] Thomas Weide,[2] and Jan Peter Siebrasse[1,*]
[1]Clausius Institute of Physical and Theoretical Chemistry, University of Bonn, Bonn, Germany; [2]University Hospital of Muenster (UKM), Internal Medicine (MedD), Muenster, Germany; and [3]Forschungszentrum Jülich GmbH, Institute of Biological Information Processing (IBI-2), Mechanobiology, Jülich, Germany

ABSTRACT Mammalian Crumbs proteins 2 (CRB2) and CRB3 are expressed in the kidney, with CRB2 mainly found within podocytes. Here, we focused on the function of CRB2 in podocytes. We investigated the CRB2/CRB3A membrane dynamics at the podocyte-podocyte contact interface in a cell culture model. Traction force microscopy was employed to clarify whether the homophilic CRB2 interactions occur in *cis* or *trans*. Live-cell imaging revealed the effects of CRB2 and CRB3A expression on cell migration from the single-cell level to large cellular networks at long space and timescales. We found that CRB2 interacts in *trans*, but surprisingly does not form stable interconnections. Instead, CRB2 acts as a dynamic sensor for the cellular environment and triggers a massive reorganization of focal adhesions and the actin cytoskeleton upon contact of CRB2-positive cells resulting in a strongly increased cell-matrix anchoring. Cytoskeleton reorganization can also be triggered by CRB3A; however, the increased cell-matrix anchorage is CRB2 specific.

SIGNIFICANCE Crumbs2 is an essential protein that has an important role in epithelial development, tissue organization and plays a decisive role in certain nephrological diseases. CRB2 is expressed in special cells of the kidney, the podocytes, but its exact function there was not yet known. We show that CRB2 senses the presence of CRB2 on neighboring cells and upon contact initiates a massive fixation to its extracellular matrix. Thereby, CRB2 is not a static component of the glomerular filtration barrier as often believed, but instead acts as a very dynamic sensor for the podocyte cell environment. CRB2 acts in *trans* to recognize other CRB2-expressing podocytes and CRB2-CRB2 interactions result in a massive reorganization of the cytoskeleton.

INTRODUCTION

The Crumbs complex is an evolutionarily highly conserved protein complex consisting of four core proteins: the eponymous single-pass membrane protein Crumbs and three intracellular adapter proteins PALS1, PATJ, and LIN7c (1–3). Crumbs complexes play a central role in apicobasal cell polarization and cell-cell junction formation, particularly in epithelial cells. In contrast to *Drosophila*, mammals express three Crumbs isoforms in cell-type- and tissue-specific patterns: CRB1, CRB2, and the two CRB3 splice variants CRB3A and CRB3B (1–3).

CRB1 plays a central role in the retina, and some mutations in the human CRB1 gene lead to retinal diseases such as retinitis pigmentosa or Leber's congenital amaurosis (for review, see (4)). CRB2 is expressed during early embryogenesis (5,6), in the retina (7), brain (8,9), and kidney (10–14). Together with CRB1, CRB2 is critical for retinal development and maintenance (4,15). Mammalian CRB3 isoforms are the most prominent Crumbs isoforms in the kidney as well as in almost all other epithelial tissues (11,16,17). *Drosophila* Crumbs and mammalian CRB1 and CRB2 proteins have large extracellular domains (ECDs) consisting of multiple epidermal growth factor (EGF)-like and three central laminin globular (G) domains, and all CRB proteins contain a small, short intracellular domain (ICD) with FERM (from: 4.1/Ezrin/Radixin/Moesin) and PDZ (from: PSD-95/Discs-large/ZO-1) binding motifs (2).

CRB2 is "reexpressed" during nephrogenesis in CRB3-positive renal progenitor cells, which later mature into postmitotic glomerular epithelial cells (10). These cells—also known as podocytes—form highly branched, interlocking

Submitted January 28, 2025, and accepted for publication July 17, 2025.
*Correspondence: jpsiebrasse@uni-bonn.de
Editor: Alexandra Zidovska.
https://doi.org/10.1016/j.bpj.2025.07.020
© 2025 The Author(s). Published by Elsevier Inc. on behalf of Biophysical Society.
This is an open access article under the CC BY license (http://creativecommons.org/licenses/by/4.0/).

cell protrusions called foot processes (FPs) to form the slit diaphragms (SDs). The SDs are unique podocyte cell-cell contacts of the glomerular filtration barrier that prevents the loss of serum proteins into the urine (for review, see (18–20)). Podocyte depletion or SD damage is associated with most forms of severe proteinuric diseases, which in turn precede renal failure (20). At the molecular level, the roles of SDs are mediated by multiprotein complexes that contain a central single-pass type I membrane protein that bridges the distance between FPs and achieves their intracellular binding.

For a long time, it was assumed that the really important part of Crumbs proteins was the ICD, which was therefore often considered the “business end” (2). Meanwhile it is clear that the huge ECD of Crumbs plays also an important role in cell-cell interactions. The main function of the ECD of Crumbs is the formation of homophilic interactions in *cis* or *trans*, i.e., within the same or between different cells, respectively. In the formation of apicobasal polarization, homophilic Crumbs interactions in *cis* play an important role, whereas in the formation of tubes in fly embryos or the morphogenesis of the retina in zebrafish, homophilic interactions of Crumbs in *trans* take place. For crb2a and crb2b in zebrafish it could be shown that this *trans* interaction is very stable and can even be used biochemically to pull down the protein domains involved (2,21). However, the exact role of the mammalian homologs CRB2 and CRB3A in human podocytes is still poorly understood.

In this study, we investigated these two proteins in an immortalized podocyte cell line, which simply served as a two-dimensional (2D) podocyte-specific tissue model (22). Using fluorescence recovery after photobleaching (FRAP), we analyzed the CRB2 and CRB3A membrane dynamics and in particular their real-time behavior at the podocyte-podocyte contact interface in vitro. Traction force microscopy was employed to clarify whether the homophilic CRB2 interactions occur in *cis* or *trans* configuration and if they contribute to stabilize cell-cell contacts. Live-cell confocal imaging was used to investigate the effects of constitutive CRB2 and CRB3A expression on podocyte growth from single-cell level to larger cellular networks at longer space and timescales. In summary, we found that CRB2 interacts in *trans*, and functions as a dynamic sensor for the cellular environment to recognize other CRB2-expressing cells. When CRB2-expressing cells come in contact, a massive restructuring of their actin cytoskeleton and especially the focal cell adhesion points of the podocytes takes place. As a result, the podocytes stably “settle down,” i.e., they anchor more firmly to the substrate as opposed to cells not expressing CRB2. While increased cell clustering and actin reorganization can also be triggered to a certain amount by CRB3A, the modulation of focal cell adhesion and increased substrate anchorage is unique to CRB2.

MATERIALS AND METHODS

DNA cloning

The pENTR plasmids carrying cDNA inserts encoding for codon-optimized or wild-type (wt) human CRB2 wt (amino acids [aa] 1–1285) with GFP-, SNAP-, or HaloTag within the 13th EGF-like repeat of the ECD (inserted after an aspartate at position 1095) have been described earlier (14). In addition, we used cDNAs expressing a CRB2 deletion mutant lacking aa 108–1094 (CRB2 ΔECD), and mutant lacking the three laminin G repeats in the middle of CRB2 ECD (CRB2 ΔLamG1-3, aa 431–1047). Further we applied the previously established expression cassettes encoding for a GFP-tag in ECD of CRB3A (CRB3A-GFP) or for a truncated form of CRB3 lacking the entire CRB3 ICD (CRB3ΔICD) (23). The pENTR plasmids were shuttled into Gateway-compatible pINDUCER21_puro or pQCXIP expression plasmids by using LR Clonase according to the manufacturer’s instructions as described earlier.

Cell culture and generation of stable cell lines

Human immortalized podocytes (AB8/13) (22) were cultivated as described earlier. Stable cell lines were generated as previously described (14). In brief, HEK293T cells were transiently transfected with psPAX2 and pMD2.G helper plasmids and the modified pINDUCER21_puro or pQXCIP expression plasmids, respectively. VSV-G-pseudo-typed virus particles containing supernatants were collected and filtered through a 0.45 μm sterile filter (EMD Millipore; Burlington MA, USA, no. GSWP01300) and added to AB8/13 podocyte cells. After 24 h the virus-particle-containing medium was replaced by fresh medium and cells were regenerated for further 24 h. Transduced AB8/13 cells were selected by puromycin (2 μg/mL; Thermo Fisher Scientific; Waltham MA, USA, no. J61278.MB). All established stable cell lines were tested for inducible overexpression of CRB2 proteins by western blot and live-cell imaging.

AB8/13 podocytes either expressing CRB2 (or not) were cultured in standard RPMI 1640 medium (Sigma-Aldrich; St. Louis, MO, USA, no. R8758) supplemented with 10% FBS superior (Bio&SELL; Nürnberg, Germany, no. FBS.S0615), with 1% PenStrep (100× solution; Sigma-Aldrich, no. P4333), 1% Amphotericin B solution (250 μg/mL solution; Sigma-Aldrich, A2942), and 0.8% supplement solution. The supplement solution was prepared by dissolving 50 mg insulin-transferrin-sodium powder (Sigma-Aldrich, no.11074547001) in 5 mL sterile water (Sigma-Aldrich, no. W3500) and adding 5 mL NEAA 100× (Gibco; Waltham, MA, USA, no. 11140-035), 5 mL Na-Pyruvate 100 mM (Sigma-Aldrich, S8636-100ML), 25 mL HEPES 1 M (Sigma-Aldrich, no. H0887-100ML). Aliquots (10 × 4 mL) were prepared and used for 500 mL medium. Cultivation was performed at 33°C in a humidified atmosphere containing 5% CO_2. Cell culture medium was replaced every 2–3 days. At a cell density of 80–90%, cells were subcultured by incubation with 0.05% trypsin-EDTA solution (Sigma-Aldrich, no. 15400054) at 33°C for 5 min. Before cell seeding, different substrates were coated with 0.01 mg/mL fibronectin (human; Corning; St. Louis, MO, USA, no. CLS354008) diluted in 1× PBS at 33°C for 30 min and subsequently washed three times with 1× PBS. For cell force measurements and immunocytochemistry staining, podocytes were plated on various cell culture substrates in a density of 4.2×10^3 cells/cm^2 in 500 μL medium (details about substrates below). All analyses were performed 24 h after cell seeding.

Live-cell imaging

Approximately 80,000 GFP tagged CRB2 wt and CRB3ΔICD cells were co-cultured with 80,000 mCherry tagged AB8/13 podocytes to attain a confluency of ∼60–70% in an Ibidi plate (Ibidi; Gräfelfing, Germany, no. 81156). A total of 25 tiles spanning ∼1.5 × 1.5 mm with a 20% overlap along with averaging twice were acquired with a 40× water immersion

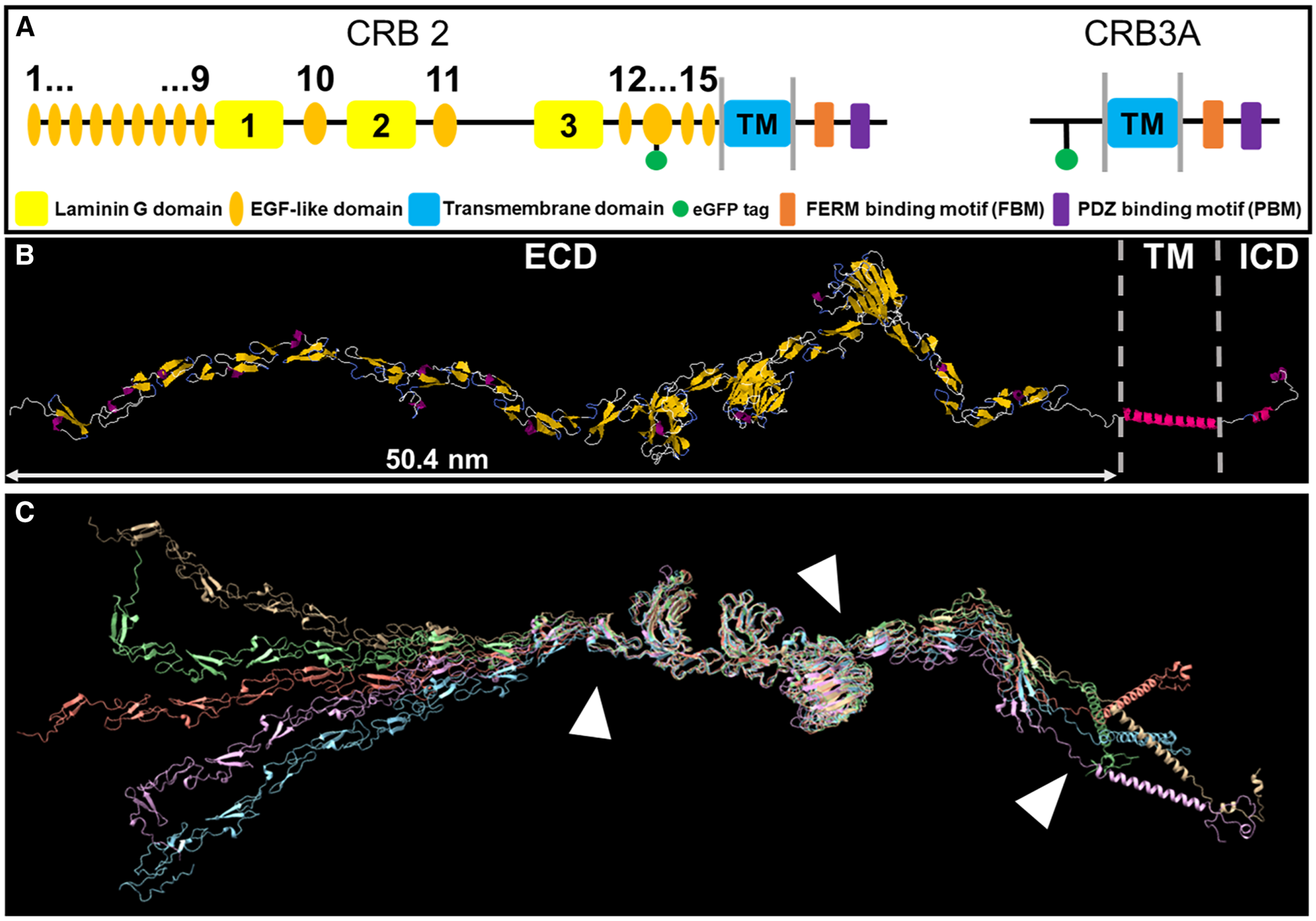

FIGURE 1 In silico prediction of the CRB2 protein structure. (*A*) Scheme of CRB2 (*left*) and CRB3A (*right*). Lamin G domains are shown as yellow rectangles, EGF-like as orange ellipses. TM, transmembrane domain. The green sphere indicates the position of the GFP in the fusion proteins used in live cell experiments. (*B*) Single computational model of CRB2 wt computed with the RoseTTaFold software (32). (*C*) Overlay of five different computational models of human CRB2 wt computed by the RoseTTaFold software indicating regions of more flexible protein folding (N- and C-terminus) and the static conserved regions of the laminin G domains. Overlay created by aligning best pair of matching chains using ChimeraX 1.8 (33). Arrowheads point to the flexible hinge-like regions.

(WI) objective with a numerical aperture (NA) of 1.2 using a Zeiss LSM 880 every 15 min for 15 h. The final image was then stitched using the image stitching function of the LSM software (Zen Black, Zeiss; Oberkochen, Germany).

To analyze the subcellular protein distribution, five Z stacks were acquired as 16-bit images for each cell type, namely, mCherry, CRB3ΔICD, CRB3A wt, and CRB2 wt. Each Z stack was acquired using the 40×, WI objective, NA 1.2 on a Zeiss LSM 880 with the Airyscan detector set to super-resolution mode. A maximum intensity projection was generated from these Z stack images. Subsequently, a circular region of interest (ROI) of 2 μm diameter was either positioned within the cell-cell membrane interface or the free plasma membrane regions. The ROIs were used to measure the mean intensity values within these specified regions. A total of three ROIs were positioned for each of the two regions specified above and the measurement was repeated for each Z stack image. Therefore, we generated a total of 15 mean intensity values for each region. Finally, from one Z stack image, we calculated the ratio between the average of three mean intensities generated from the overlapping region ROIs divided by the average of the three mean intensities generated from the free plasma membrane region ROIs. This calculation was repeated for each Z stack image giving us 5 ratio values/replicates for each cell type.

For co-cultivation of GFP/HaloTag-expressing cells, living AB8/13 podocytes expressing CRB2-WT-HaloTag and CRB2-WT-eGFP were seeded simultaneously into Ibidi plates for live-cell microscopy at the desired cellular density of ~40% confluency. HaloTag staining with JaneliaFluor646 (Promega; Madison, WI, USA, no. GA1120) was performed as per the manufacturer's instructions. First, co-cultured cells were washed three times with PBS pre-warmed to 33°C. JaneliaFluor646 was diluted with pre-warmed growth medium from a 200 μM stock solution made with DMSO (Sigma-Aldrich, no. D2650) down to a final concentration of 4 μM. Cells were subsequently incubated with the diluted dye medium mixture for 1 h at 33°C with 5% CO_2. Finally, cells were washed three times with pre-warmed normal growth medium for 10 min each before transferring the plate to the microscope.

FRAP measurements

GFP-expressing cells were seeded in Ibidi plates (Ibidi, no. 81156) such that the plates achieved 70% confluency on the day of measurement. Cells expressing the inducible constructs were cultured in media supplemented with 200 nM doxycycline 2 days before the FRAP measurements with medium change every 24 h to achieve optimal expression. Similarly, cells expressing the constitutive constructs were subjected to normal medium change every 24 h. Based on the localization of the signal, two distinct circular ROIs were chosen (Ø 2 μm), namely, within overlapping cell membrane duplets or within the free plasma membrane. Three plates from three different days were chosen to take measurements for every ROI from each cell type. The GFP signal in the ROIs was bleached with 3 iterative scans with 488/405 nm irradiation (100% transmission; overall bleaching time 75 ms) and the recovery recorded for 30 s with 150 ms/frame followed by 90 s recording with 1 s/frame only with the 488 nm laser line (<1–2% transmission). A minimum of 30 measurements were recorded from at least 15 different cells harboring at most 2 ROIs of the same type from every plate. The plate triplicates, therefore, provided for at least 90 FRAP measurements for each ROI from every cell type. These measurements were

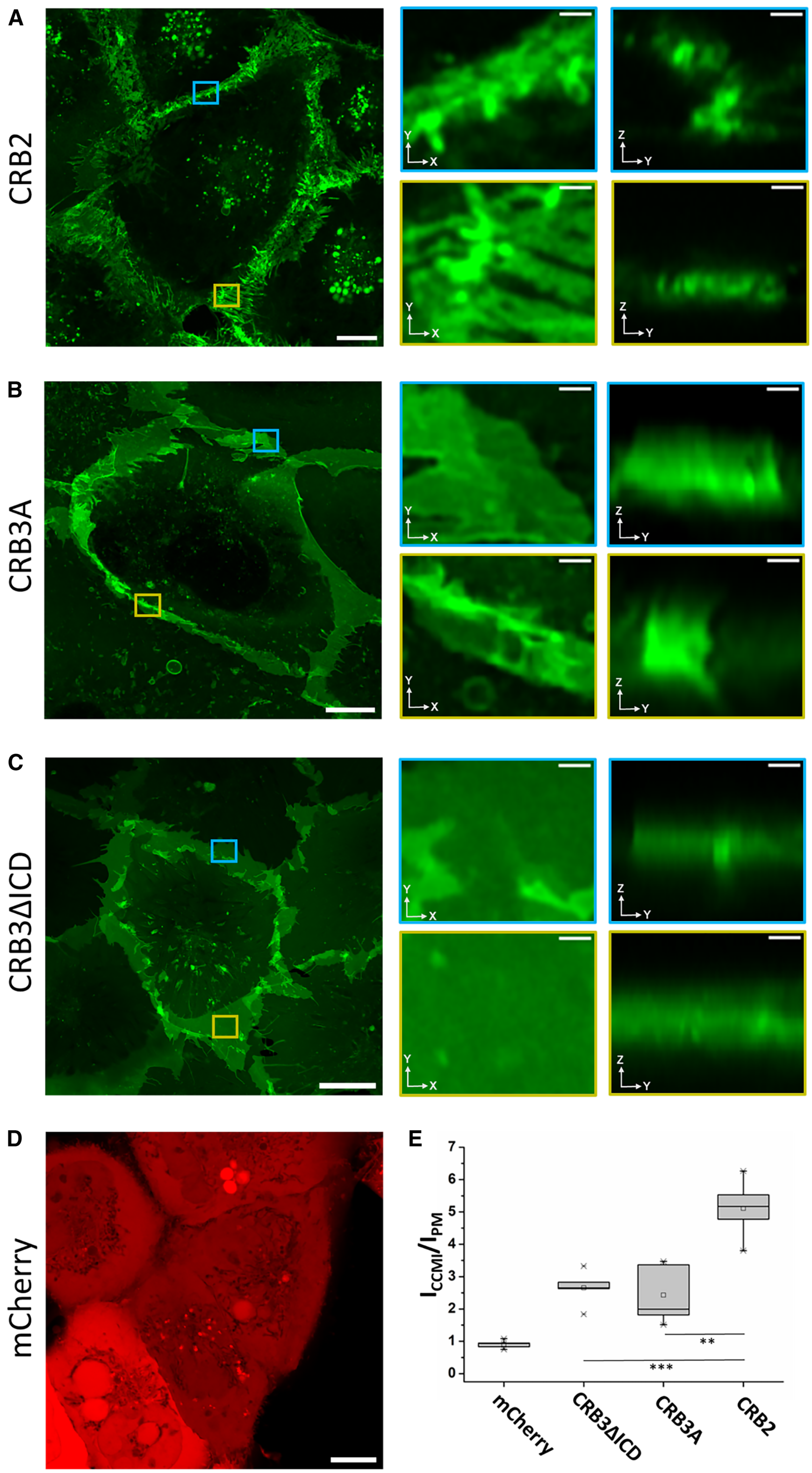

FIGURE 2 Subcellular localization of CRB2 wt, CRB3A wt, CRB3ΔICD GFP fusion proteins, and mCherry in cultured AB8/13 podocytes. (*A*) CRB2-wt-GFP, (*B*) CRB3A-wt-GFP, (*C*) CRB3ΔICD-GFP. The small images on the right-hand side show a zoomed-in view of the, respectively, marked regions on the left-hand side. Scale bars, 20 μm (left) and 1 μm (right zoom-ins). (*D*) mCherry distribution inside an AB8/13 cell. (*E*) Ratio of the fluorescent protein signal at the cell-cell membrane interface I_{CCMI} and the free plasma membrane I_{PM}. Significance $***p < 0.001$, $**p < 0.005$. The crosses indicate the maximum range of the values.

performed at 33°C with 5% CO_2 with the Zeiss LSM 880 using the 40× WI objective lens, NA 1.2, and fully opened pinhole. Initial testing with doxycycline concentrations indicated that protein expression is typically initiated after 12 h. Hence, FRAP measurements from each plate were strictly completed within 6 h. ROIs for bleaching and background correction were carefully positioned to avoid intracellular vesicles within or close to the ROIs (see Fig. S3). Each FRAP measurement was evaluated individually after recording. Measurements with transport vesicle intrusions or cell

movements were discarded. For details on the data acquisition process, data normalization, and fitting process (see Fig. S3).

Growth pattern analysis

Approximately 20,000 AB8/13 podocytes constitutively expressing either GFP fusions of CRB2 wt, CRB3A wt, or CRB3ΔICD were mixed with 20,000 AB8/13 podocytes expressing mCherry to achieve a confluency of ~20% in an Ibidi plate (Ibidi, no. 81156). The cells were then cultured for 7 days with medium change every 2 days. At the end of the week, cells were fixed with 4% (w/v) paraformaldehyde (Electron Microscopy Sciences; Hatfield, PA, USA, no. 15714-S) to preserve GFP and mCherry fluorescence and nuclei were counterstained with DAPI. The whole-cell culture dish observation area with a diameter of 21 mm was then imaged with a 10× air objective NA 0.8 on the Zeiss LSM 880 (pixel size ~2.8 μm) using the GFP, mCherry, and DAPI signal. A total of 400 tiles covering approximately 23 × 23 mm with a 20% overlap and two-fold frame averaging were acquired from each co-cultured plate. The final image was then stitched using the image stitching function of the LSM software. For subsequent image analysis FIJI (24) was used. The GFP channel was used to create a binary image by manual intensity thresholding, and the binary image was eroded and dilated again one time to smoothen the binary masks. FIJI's particle analysis function was used to identify individual cell clusters, and the perimeter of each cluster was calculated for all identified clusters. The perimeter of the largest cluster found in the CRB3ΔICD control served as a threshold for the occurrence of random clusters, and all clusters with perimeters smaller than the threshold were removed from the CRB2 wt, CRB3A wt, and CRB3ΔICD images. Using the respective binary mask and the nuclear DAPI signal, the number of cells in clusters versus all cells in the dish could be determined for quantification. All image analysis was done on a dedicated high-speed server (Hive, Bruker Corporation; Billerica, MA, USA) equipped with 1 TB RAM and a 48 Kernel CPU.

Immunofluorescence staining

For immunocytochemistry staining, podocytes either expressing Crumb2 or not were plated on 35-mm glass-bottom μ-dishes (growth area = 3.5 cm^2, Ibidi, no. 81156). After cultivation for 24 h, podocytes were fixed with 2% PFA (v/v) (Thermo Fisher Scientific no. J19943.K2) and 1% glutaraldehyde (v/v) (Merck; Darmstadt, Germany, no. G7776-10ML) diluted in 1× cytoskeleton buffer (CB) (5 mM EGTA [Sigma-Aldrich, no. E3889], 5 mM glucose [Sigma-ldrich, no. G8270-100G], 10 mM MES [Sigma-Aldrich, no. M3671], 10 mM $MgCl_2$ [Sigma-Aldrich, no. M9272], 150 mM NaCl [Sigma-Aldrich, no. S5886], 1.72 mM streptomycin [Sigma-Aldrich, no. S9137] diluted in ultrapure water) for 30 min at room temperature (RT). Permeabilization was performed using 1% Triton X-100 (v/v) (Sigma-Aldrich, no. X100-5ML) diluted in 1× CB for 5 min at RT. Afterward samples were washed twice with 1× CB for 5 min at RT. Then, unspecific antibody binding sites were blocked with blocking buffer (1× CB supplemented with 0.1% [v/v] BSA [Sigma-Aldrich, no. A7906], 0.2% [v/v] Triton X-100 [Sigma-Aldrich, no. X100-5ML], 0.05% [v/v] Tween 20 [Sigma-Aldrich, no. P9416-50ML] containing 5% skim milk (m/v) (Carl Roth; Karlsruhe, Germany, no. 68514-61-4) for 4 h at RT. Again, all samples were washed twice with 1× CB for 5 min at RT. Primary anti-vinculin hVin1 antibody (Sigma-Aldrich, no. V9131-100UL) was diluted 1:500 in blocking buffer containing 1% (m/v) skim milk (Carl Roth; Karlsruhe, Germany, no. 68514-61-4) and was then incubated for 16 h at 4°C. After incubation, samples were washed twice with 1× CB for 5 min at RT. Secondary antibody Alexa Fluor 488 goat anti-mouse IgG (Thermo Fisher Scientific, no. A11001) and Alexa Fluor 546 Phalloidin (Thermo Fisher Scientific, no. A22283) were diluted in 1:500 blocking buffer with 1% (m/v) skim milk and applied to samples for 1 h at RT in the dark. Washing of samples was again performed as already described. Next, nuclei were stained for 10 min at RT with two droplets of NucBlue Fixed Cell ReadyProbes Reagent (DAPI) (Thermo Fisher Scientific, no. 62248) in 1 mL 1× CB. All washing and incubation steps were performed on a 2D shaker, and samples were stored at 4°C until imaging.

Stained samples were imaged at RT with an inverse confocal laser scanning microscope (LSM880 with Airyscan detector, Zeiss) that used a 63× Plan-Apochromat oil immersion objective (NA 1.4, Zeiss). Excitation lasers were a diode laser (405 nm), an argon-ion laser (488 nm), and a diode-solid-state laser (561 nm). All images were taken and processed with the same settings using the Zen 2.3 black software (Zeiss) with a scaling of 0.11 × 0.11 μm per pixel for cell duplets and 0.06 × 0.06 μm for single cells.

Quantification of focal adhesions and the actin cytoskeleton

Podocyte duplicates with and without CRB2 wt were grown on glass substrates for 24 h, fixed and stained for the indicated proteins (Figs. S6 and S7). Focal adhesions (FAs) (cell matrix contacts) were stained for vinculin, imaged with confocal microscopy (see above; Fig. S6 *A*), and analyzed with regard to their number, size, and degree of coverage. To detect FAs, the local z-score was used to separate the bright FAs from the background (Fig. S6 *B*) as described in (25). FAs were only analyzed within the cell mask, created from actin staining as described below (Fig. S6, *C* and *D*).

Peripheral actin distribution was analyzed by automated cell segmentation using the following procedure. Cells were separated from background by using the mean gray value of the image as intensity threshold. This segmented cell mask was then post processed by morphological closing, filling holes in the mask, removing objects connected to the image border, and finally choosing the biggest label as cell or cell duplet mask. Average intensities of actin staining were calculated within this mask; total mask size served as area of cell or cell duplet, respectively. Actin intensities were analyzed in one-pixel-wide rings from outside to inside (Fig. S6, *C* and *D* show the outermost ring; pixel size 0.11 μm for cell duplets and 0.06 μm for single cells). To this end, the distance transformation of the cell duplet mask was calculated and gray values of all pixels with the same distance to the mask border were averaged. Finally, these averaged gray values were normalized by the mean gray value of the whole cell or cell duplet, respectively.

Wound healing assay

To analyze the migratory behavior of cells, wound healing assays were performed. Cells were seeded in an Ibidi imaging dish (35 mm, Ibidi, no. 81156) with a two-chambered silicon insert and cultured until confluency. The silicon insert was removed and cells could migrate into the cell-free gap over 24 h. Images of the scratch were captured in the beginning and in regular intervals during the experiment via microscopy in a 10× magnification. The quantitative analysis was performed using the MRI Wound healing tool of ImageJ. Based on a defined variance filter radius applied in this tool, the cell-free zone is separated from the cell-containing zone and the wound area is calculated.

Traction force microscopy

For cell force measurements, AB8/13 podocytes either expressing Crumb2 or not were grown for 24 h on cross-linked silicone (Sylgard 184, 15 kPa; mixing ratio of base oil to cross-linker 55:1 per weight; Dow, Midland, MI, USA). Substrates were prepared as described in Hersch et al. (25), micro patterned with fluorescent beads (FluoSpheres carboxylate, 0.2 μm, crimson (625/645); Thermo Fisher Scientific) as described in (26), and coated with fibronectin (as described above) before cell seeding. Cell growth area was 2.54 cm^2.

Live-cell imaging for traction force microscopy (TFM) analysis was performed at 33°C and 5% CO_2 (cell incubator XL, Zeiss) with a wide-field microscope (Axio Observer, Zeiss) equipped with an AxioCam MRm

camera (Zeiss). A 40 EC Plan-Neofluar oil immersion objective (Ph3, NA 1.3, Zeiss) was used in combination with a multicolor LED light source (Colibri 7, Zeiss). Images were taken using the ZEN 2.3 blue edition software (Zeiss) with a scaling of 0.345 × 0.345 μm per pixel.

Single cells and cell duplets were imaged using phase contrast to localize cells and fluorescence microscopy to visualize beads. Tangential substrate deformations caused by traction forces were quantified via bead displacements. To obtain the displacement vector field, single cells and cell duplets were first imaged. Then cells were removed by trypsin digestion and the same substrate positions were imaged again. Beads were localized and their displacements calculated by algorithms described in (27). Maps of cell-induced traction stresses were calculated by regularizing last-square fitting to the mechanical response of an elastic layer of 80 μm thickness on rigid substrate, here glass (27,28). From these maps, strain energy was calculated as proposed by (29). Strain energy is the mechanical energy stored in the deformed substrate and a convenient scalar measure of the overall mechanical activity based on previous work. Strain energy integration was done in a region enclosing cell (or duplet) and its close vicinity.

Statistical analysis of the FRAP curves

The τ_1 time constants and maximum recovery values were calculated for each single FRAP curve and the values were plotted as boxplots using Eq. 1 and Eq. 2 from Fig. S3 (see also Fig. S4). A two-way ANOVA with repeated measures was employed to test for significant differences 1) for the different proteins in the same membrane localization, i.e., either all CRB2/3 in the "free plasma membrane" or all CRB2/3 in the "cell-cell membrane interface" or 2) for differences for the same protein in the two membrane domains, e.g., difference between CRB2 wt in "free plasma membrane" versus CRB2 wt in the "cell-cell membrane interface." Means comparisons were done using the Tukey test with $\alpha = 0.05$, $\alpha = 0.01$, and $\alpha = 0.001$. To compare the quantitative measurements in the growth pattern analysis, a two-sample t-test was used for pairwise testing of CRB2wt, CRB3Awt, or CRB3ΔICD. All statistical calculation were performed using OriginPro 2021b (30).

RESULTS

In silico prediction of the CRB2 protein structure

CRB2 is a huge transmembrane protein comprising of 1285 residues with an overall molecular mass of ∼134 kDa. It contains 15 EGF-like domains mostly organized in 2 EGF-repeat clusters at the N- and C-terminal positions of the ECD (Fig. 1 *A*).

Between the two EGF-repeat domains three central laminin G domains are located, separated from each other by a single EGF-like domain. The ICD is rather short and contains the FERM and PDZ binding motifs (FBM/PBM). The transmembrane domain and the ICD of CRB2 and CRB3A are highly similar but not identical and CRB3A is missing a significant ECD (Fig. 1 *A*, *right scheme*). So far, full-length CRB2 has never been crystalized for x-ray structural analysis. Therefore, we used computational protein modeling approaches to predict the molecular arrangement of the different domains. The signal peptide cleavage position was provided by Signal-P (31). Amino acid sequences post cleavage were then fed to protein structure prediction algorithms. We used the RoseTTaFold molecular modeling software (32) and got from five rounds of simulations five very comparable structures (Figs. 1 *C* and S1), a single one is shown in Fig. 1 *B*. The resulting structures always show an elongated molecule with five distinct domains. In the ECD, the large N-terminal EGF-repeat is followed by the laminin G domains and the smaller membrane-proximal EGF-repeat. Inside the cytosol the very flexible ICD with the FBM and PBM motifs are located and both are connected to the ECD via the transmembrane domain. The error estimates as functions of the amino acid position reveal that the structures show strong positional variations at the N- and C-terminal EGF-repeats, while the central laminin part can be estimated repeatedly with a very small error (<5 Å, *yellow rectangle* in Fig. S1). The five structures were superimposed using the Matchmaker function from ChimeraX 1.8 (33) to emphasize the more flexible versus static subdomains (Fig. 1 *C*). While the central laminin G domains always fold computationally into a very similar structure, the adjacent EGF domains appear more flexible and bent around two hinge-like regions between the three ECD domains. The orientation of the entire ECD to the transmembrane α-helix domain also appears to be very flexible (see *arrowheads* in Fig. 1 *C*). Depending on the respective bending angle the overall length of the ECD varies from 40.1 to 50.4 nm (Figs. 1 *C* and S1). In summary, the in silico folding calculations indicate that CRB2 is not a rigid rod-shaped molecule, but is more flexible in its overall molecular conformation. Eventually, the selectable orientation of the extracellular part of CRB2 can be modulated by homophilic interactions in *cis* or *trans*. To analyze the potential influence of the ECD and ICD on the subcellular localization of CRB2 and CRB3A we expressed them and respective mutant constructs as GFP fusion proteins. To ensure that any observed effects of CRB2 wt and mutants cannot ultimately be caused by overexpression of the respective proteins, the expression level of the different proteins in the inducible and constitutively expressing cells was checked using western blots (Fig. S2).

Subcellular localization of CRB2 in cultured podocytes

GFP fusion proteins of CRB2 wt (Fig. 1 *A*, *left scheme*) and CRB3A wt (Fig. 1 *A*, *right scheme*), and its deletion mutant CRB3ΔICD comprising only the transmembrane domain and the extracellular GFP, were expressed in the human podocyte cell line AB8/13 (22). These cells do not intrinsically express CRB2 but only CRB3A at a low transcriptional level (14). The subcellular distribution of the proteins was visualized by confocal imaging in living cells. In particular, protein localization within cell-cell membrane interfaces of podocytes in direct contact was investigated. To this purpose, cells were grown to almost confluent cell density (60–70%). Since CRB2 wt, CRB3A wt, and CRB3ΔICD contain a transmembrane domain, they are almost exclusively localized to the plasma membrane of the AB8/13 podocytes (Fig. 2, *A–C*) compared with the podocytes expressing an unrelated

fluorescent protein without membrane specificity (mCherry; Fig. 2 *D*). However, the protein signal differs in the plasma membrane at cell-cell interfaces for CRB2 wt, CRB3A wt, and CRB3ΔICD. CRB3A wt and CRB3ΔICD appear to be evenly distributed throughout the cell with slightly brighter fluorescent signals in the membrane contact regions due to the axial overlap of the four plasma membranes (Fig. 2, *B* and *C*). On the other hand, CRB2 displays a more anisotropic distribution and appears more enriched at these overlapping regions while the intensity in the free plasma membrane seems comparably lower (Fig. 2 *A*). To analyze the protein distribution more precisely, Z stacks were acquired for each cell type, and from their maximum intensity projections the fluorescence intensity in the cell-cell membrane interface (I_{CCMI}) or the free plasma membrane regions (I_{PM}) were measured. From this, the intensity ratio between the two membrane compartments was calculated for each protein (I_{CCMI}/I_{PM}; Fig. 2 *E*). As expected, the soluble control protein mCherry showed an even distribution between the free plasma membrane and the cell-cell contacts ($I_{CCMI}/I_{PM} \sim 1$), while CRB3A wt and CRB3ΔICD showed an increased intensity at the cell-cell contacts due to the stacked plasma membranes. However, the signal ratio of CRB2 wt was significantly higher ($I_{CCMI}/I_{PM} > 5$; Fig. 2 *E*), which implied that the protein was clearly more enriched at the cell-cell membrane interface compared with CRB3A wt and CRB3ΔICD. To clarify whether this accumulation was possibly due to local immobilization of CRB2 wt at the cell-cell membrane interfaces, we examined the mobility of CRB2 wt against various CRB2 mutants, and compared it with the corresponding mobility of CRB3A wt and CRB3ΔICD.

Membrane dynamics of CRB2 in cultured podocytes

FRAP experiments represent a classical approach to examine the mobility behavior of membrane proteins in terms of diffusion speed and mobile or immobile fractions (34). We performed FRAP measurements of CRB2 wt, various CRB2 ECD/ICD mutants, and CRB3A wt and CRB3ΔICD, which were all expressed as GFP fusion proteins in AB8/13 podocytes (see Fig. 1 *A*). The cells were maintained on the microscope stage of an LSM880 (Zeiss; Oberkochen, Germany (35)) during FRAP measurements under the same conditions as during cell culture. All measurements were taken under the very same experimental conditions and within the same time window, i.e., 48 h after inducing protein expression. To analyze possible mobility differences in different membrane regions we carried out measurements both in the area of the cell-cell membrane interface of directly neighboring cells (Fig. S3, *A* and *B*) and in the free plasma membrane (Fig. S3, *C* and *D*; see also supporting material). To understand the influence of the different ICD and ECDs, we generated respective deletion mutants of CRB2 wt and included CRB3A wt and CRB3ΔICD for comparison. The aforementioned CRB3ΔICD is a mutant without the ICD and consists practically only of the transmembrane domain and the extracellular GFP. For CRB2 we used three mutants. In CRB2ΔPBM and CRB2ΔFBM, the respective intracellular PBM and FBM domains were either deleted or inactivated by mutation. In the CRB2ΔECD mutant, almost the entire ECD was removed except for the first and last three membrane-proximal EGFs, as the complete removal of the ECD resulted in the protein getting stuck in the endoplasmic reticulum. We could not use the CRB2ΔICD mutant for FRAP measurements, because it was retained in the endoplasmic reticulum. For each GFP fusion protein a total of 90 FRAP curves was collected in 3 independent experiments. Average FRAP curves were calculated from the pooled data using Eq. 1 from Fig. S3 and shown in Fig. 3, *A–F*.

These FRAP curves immediately revealed general mobility patterns. A rapid and almost complete maximum recovery of CRB3A wt, CRB3ΔICD (Fig. 3 *A*), and CRB2ΔECD (Fig. 3 *C*) was observed in the free plasma membrane areas of the podocytes. A complete maximum recovery means a high fraction of mobile molecules. CRB2 wt and the ICD deletion mutants ΔFBM and ΔPBM displayed a generally slower and also a lower recovery both in the free plasma membrane and the membrane interfaces of cells in contact (Fig. 3, *B* and *E*). However, while CRB3A wt and CRB3ΔICD were faster and more complete in their recovery compared with CRB2 wt in both membrane regions (Fig. 3, *A* and *D*), the CRB2ΔECD mobility showed a dramatic change from the free plasma membrane (Fig. 3 *C*) to the cell-cell membrane interface (Fig. 3 *F*). In the free plasma membrane, CRB2ΔECD behaved almost like the highly mobile CRB3A wt and CRB3ΔICD, but in the cell-cell interface it displayed the lowest maximum recovery from all proteins examined.

To obtain a deeper insight into the differences in the dynamics of the examined proteins, we fitted a double exponential growth function to every single FRAP curve using Eq. 2 from Fig. S3 (see materials and methods and Fig. S3 (36)), and calculated mean values of all fitting results (Table 1). Fig. 3, *G–J* show boxplots of these results.

The time constant τ_1 is a parametrization of protein mobility, a small value corresponds to a fast recovery and thus high protein mobility or diffusion speed. Similarly, A_1 reports on the maximum recovery, a value close to unity indicates a high fraction of mobile molecules or a small fraction of immobile ones, and a value close to 0 indicates a small fraction of mobile molecules or a high fraction of immobility. Employing a two-way ANOVA analysis, we found that CRB2 wt and CRB3A wt showed a significant difference in their mobility. CRB3A wt was clearly more mobile within the free plasma membrane ($\tau_1 = 2.1 \pm 0.1$ s) and the cell-cell membrane interface ($\tau_1 = 2.8 \pm 0.1$ s) compared with CRB2 wt in the free plasma membrane ($\tau_1 = 4.2 \pm 0.4$ s) and in the membrane interface ($\tau_1 = 5.6 \pm 0.4$ s). There was also a significant difference with regard to their mobile

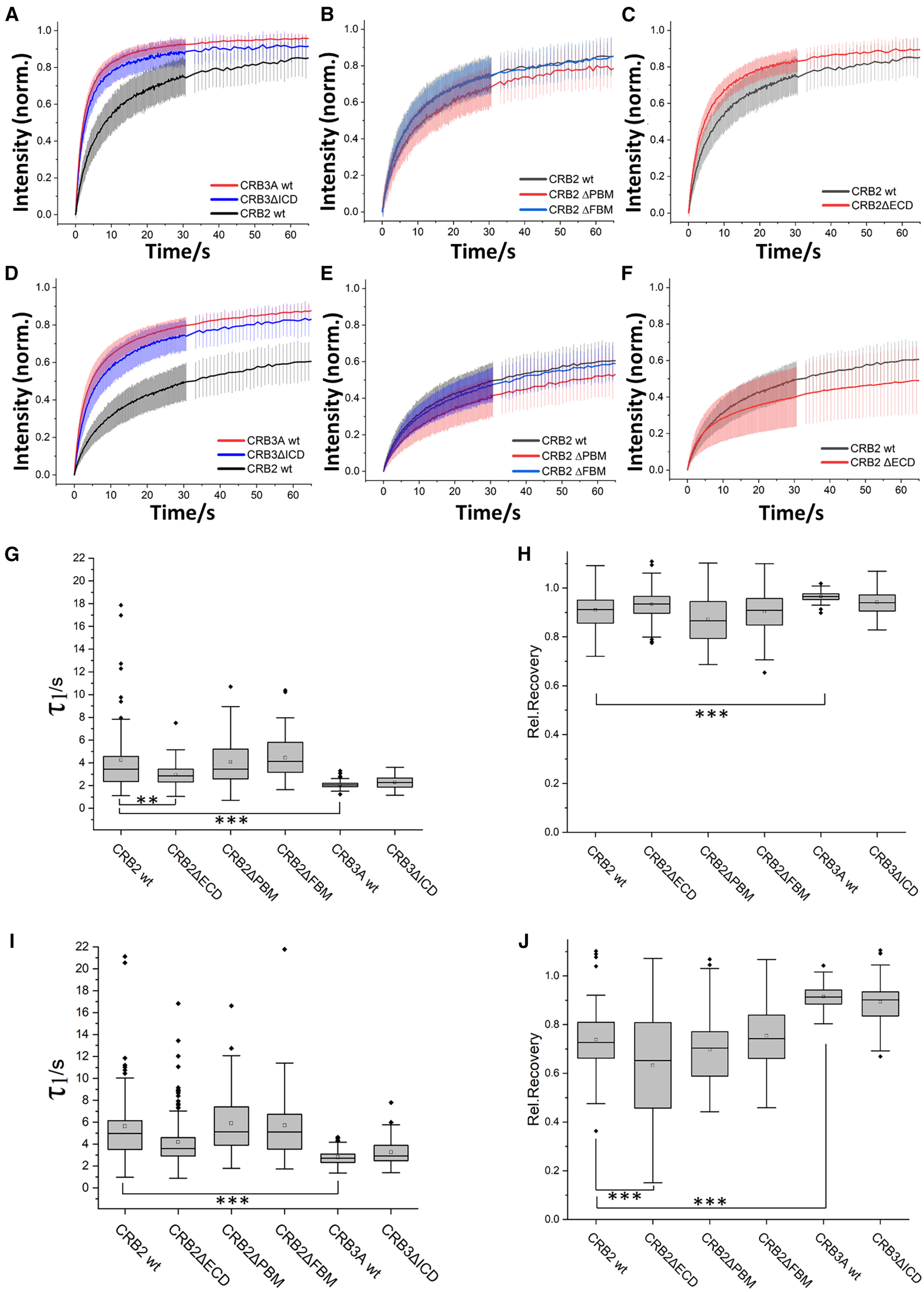

FIGURE 3 FRAP analysis of CRB GFP fusion proteins in AB8/13 podocytes. (*A–C*) Averaged FRAP curves in free plasma membrane. In each of the graphs we used the same CRB2wt data as reference. (*D–F*) Averaged FRAP curves at the cell-cell membrane interfaces. The vertical lines represent the standard deviation of the data points of the individual measurements. To facilitate visual comparison, only the first 60 s of the recovery curves are shown. As above, in each of the graphs we used the same CRB2wt data as reference. (*G*) Boxplot of time constant τ_1 along with the mobile fraction A_1 (*H*) in the free plasma membrane. (*I*) Boxplot of τ_1 along with the mobile fraction A_1 (*J*) in the cell-cell membrane interface. Significance, ***$p < 0.001$, ** $p < 0.005$.

TABLE 1 Time constants and mobile fractions

	Free plasma membrane			Cell-cell membrane interface		
Protein	τ_1 (s)	τ_2 (s)	A_1	τ_1 (s)	τ_2 (s)	A_1
CRB2 wt	4.2 ± 0.4	32.7 ± 3.2	0.91 ± 0.01	5.6 ± 0.4	55.6 ± 5.9	0.74 ± 0.02
CRB2ΔFBM	4.4 ± 0.2	37.2 ± 3.0	0.90 ± 0.01	5.8 ± 0.4	64.0 ± 4.8	0.75 ± 0.02
CRB2ΔPBM	4.1 ± 0.3	36.0 ± 3.4	0.87 ± 0.01	5.9 ± 0.4	70.3 ± 5.6	0.70 ± 0.02
CRB2ΔECD	2.9 ± 0.1	25.7 ± 1.4	0.93 ± 0.004	4.2 ± 0.2	51.0 ± 8.0	0.63 ± 0.01
CRB3A wt	2.1 ± 0.1	15.1 ± 0.5	0.96 ± 0.002	2.8 ± 0.1	25.4 ± 2.7	0.92 ± 0.005
CRB3ΔICD	2.3 ± 0.1	25.4 ± 2.7	0.94 ± 0.005	3.2 ± 0.1	31.7 ± 6.0	0.89 ± 0.01

Time constants τ_1 and τ_2 of the biexponential FRAP curve fits and normalized maximum fluorescence recovery values (A_1) for all CRB2 and CRB3 proteins tested. Each value is based on ~90 experiments, the deviation refers to the standard error of the mean.

fractions (Table 1). CRB3A wt had a high mobile fraction both in the free plasma membrane ($A_1 = 0.96 \pm 0.002$) and the cell-cell membrane interface ($A_1 = 0.92 \pm 0.005$), whereas we observed for CRB2 wt a significant decrease of the mobile fraction in the free plasma membrane ($A_1 = 0.91 \pm 0.01$) and especially within the cell-cell membrane interface ($A_1 = 0.74 \pm 0.02$; see also Fig. S4). Notably, no significant differences to CRB2 wt were observed when the PBM or FBM motifs in the ICD were removed or mutated. Rather, the time constants and also the mobile fractions were in the range of the wt protein.

The removal of a large part of the ECD of CRB2 wt had a significant impact on the protein's mobility. In the free plasma membrane, CRB2ΔECD behaved more like CRB3A wt and CRB3ΔICD with a $\tau_1 = 2.9 \pm 0.1$ s and a mobile fraction of $A_1 = 0.93 \pm 0.004$. In the cell-cell membrane interface, however, the mobile fraction of CRB2ΔECD dropped down to $A_1 = 0.63 \pm 0.01$, which was significantly lower than the mobile fraction of CRB2 wt. Interestingly, the deletion of the ECD primarily reduced rather the mobile fraction of CRB2 and less its membrane mobility. This became obvious, when we plotted the CRB2ΔECD time constants of the individual measurements against the corresponding mobility fractions in a combined scatter/box diagram (Fig. S5).

In summary, our FRAP experiments showed that CRB3A wt diffused generally faster and had a higher mobile fraction than CRB2 wt. Furthermore, we found that CRB2's membrane mobility was altered when CRB2 wt-positive cells came into contact. CRB2 became partly immobilized in these interfaces, but this did not change when either the FBM or PBM domains were missing. Finally, CRB2 without a functional ECD showed a completely different mobility pattern and was often strongly immobilized in the cell-cell membrane interfaces. To investigate whether the immobilization of CRB2 wt at the membrane contacts stabilized or enhanced the direct cell-cell interaction, we used traction force microscopy on AB8/13 podocytes.

Traction force microscopy of CRB2-expressing podocytes

The results obtained so far suggested that CRB2 apparently exerted its effects as a consequence of direct cell-cell contacts implying a *trans* mode of action. We wanted to know how CRB2 affected the cell-cell junction directly. To this end, we used traction force microscopy (29,37,38) on AB8/13 podocytes that either expressed (Fig. 4 *A*) or did not express CRB2 wt (Fig. 4 *B*) to examine the CRB2-dependent effect on the cellular force field. For these measurements, cells were seeded on soft, elastic substrates whose surface was decorated by fluorescent beads that served as markers for cell-force-induced substrate deformation. Since the GFP emission would spectrally overlap with the bead signal, CRB2 wt was expressed as a Halo fusion protein but not fluorescence stained to keep it comparable with the otherwise used GFP fusion protein.

Fluorescent images of the fluorescent beads were taken with cells intact and after cell removal by trypsinization. Bead positions were determined in both images and the cell-force-induced displacement calculated for each bead. This yielded the displacement vector field. Since the mechanical properties of the elastic substrate were well known, the spatial distribution of cell forces could be determined from the displacement vector field. Strictly speaking, the tangential stress field that the cells exerted on the substrate was determined. Please note, force retrieval requires solving a numerically ill-posed problem, which leads to similar problems that are familiar from image deconvolution (see, e.g., (27), our implementation of the algorithms is described in (28)). For a straightforward measure of overall cell force, we calculated the mechanical energy stored in the substrate from tangential stress and displacement fields as proposed by (29). Only single cells (Fig. 4 *C*) or cell duplets—either CRB2 wt positive or negative (Fig. 4 *D*)—were analyzed.

We hypothesized that interactions between the CRB2 proteins of two adjacent cells would directly stabilize cell-cell contacts. As stronger cell adhesions generally go hand in hand with increased force transfer via them, a mechanically strengthened contact between neighboring cells should result in increased cell forces that point toward the other cell. Indeed, while CRB2 wt had no significant effect on the strain energy caused by single cells (Fig. 4 *C*), duplets of CRB2 wt-expressing cells exhibited a significantly increased overall strain energy compared with CRB2

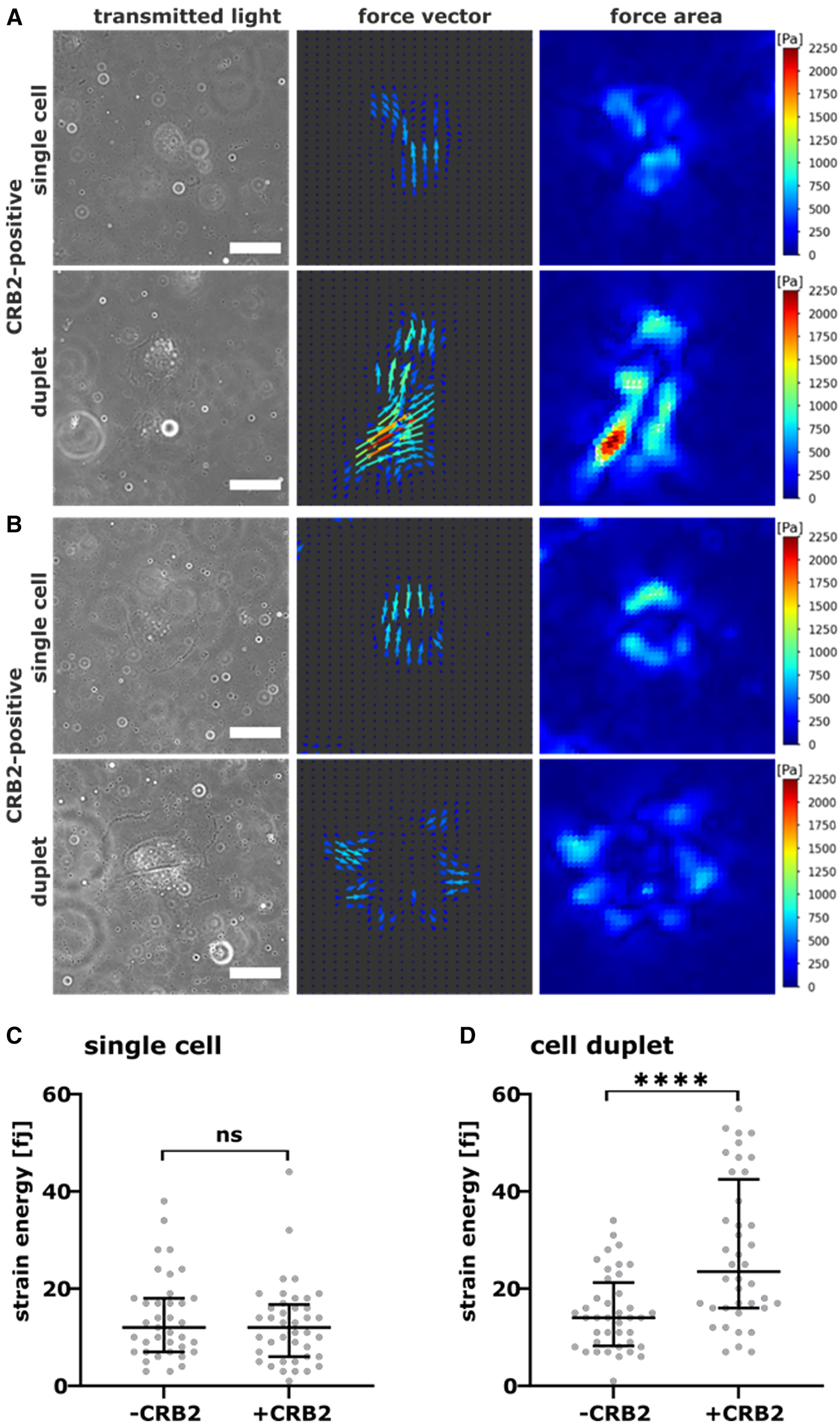

FIGURE 4 Analysis of cell-cell interactions by traction force microscopy of CRB2-positive and -negative podocytes. (*A*) Typical results for CRB2-positive AB8/13 cells. Transmitted light images (*left*), calculated forces given as vector field (*middle*), and heatmap of amplitudes (*right*). The false color code applies to both. Only single cells or cell duplets were analyzed. Scale bars, 20 μm. (*B*) The same representation as in (*A*) for CRB2-negative AB8/13 cells. (*C*) Statistical analysis of traction force microscopy results for single cells. (*D*) Statistical analysis of traction force microscopy results for single cells and duplets. Shown are individual strain energies, medians, and quartiles (all conditions, three independent samples; single cells, CRB2 37 cells; all other, >40 cells or cell duplets, respectively). Significance was tested with an unpaired *t*-test using Prism 10 (GraphPad; ns, not significant, ****$p < 0.0001$).

wt-negative cells (Fig. 4 *D*). The measured forces were indeed higher, but surprisingly the force fields did not at all point toward cell-cell contacts. In summary, upon contact, cells expressing CRB2 wt applied significantly higher forces to the substrate, but no measurable direct force transfer occurred between both cells (Fig. 4 *A*).

Immunochemistry of CRB2-expressing podocytes

To investigate this unexpected result in more detail, we performed an immunocytochemical study of the force-producing actin cytoskeleton and the FA that linked it to the

substrate (Fig. 5). CRB2 wt-expressing and CRB2 wt-negative AB8/13 cells were examined on cover glass (Fig. 5 *A*). Duplets of cells expressing CRB2 wt exhibited fewer actin fiber bundles, were less likely to show lamellipodia, and actin was more localized at the cell border in a cortical structure compared with duplets of CRB2 wt-negative AB8/13 cells (for details on quantification, see materials and methods and Fig. S6). For single CRB2 wt-expressing cells, we noted a large scatter of these features, whereas duplets were more homogeneous. In addition, CRB2 wt expression increased number (Fig. 5 *B*) and decreased size of FAs (Fig. 5 *C*) when vinculin was used as a marker. Even though FAs were fewer, they covered a larger area of the cell-substrate interface compared with the control (Fig. 5 *D*).

In cell duplets, expression of CRB2 wt increased actin intensity (Fig. 5 *E*). Moreover, with CRB2 wt, actin filaments formed a prominent cortex resulting in intensity profiles that peaked at the periphery (Fig. 5 *F*). Neither of this was observed in single cells (Figs. 5 *G* and S7). Interestingly, CRB2 wt-expressing cells in contact with each other were also significantly smaller than the corresponding AB8/13 wt cells (Fig. 5 *H*). These drastic changes of cellular morphology could not be observed in single cells (Fig. 5 *I*). The smaller size of CRB2 wt-positive cells made their overall higher strain energy even more astonishing.

To see if the changes in cell morphology were CRB2 specific, we also analyzed the impact of CRB3A wt on actin fibers and FAs. To this end we used AB8/13 cells expressing either CRB2 wt or CRB3A wt as GFP fusion protein (Fig. 6) and compared them with CRB2 wt-negative AB8/13 cells (Fig. S8). As expected, CRB2-wt-GFP showed a very similar effect on actin fibers and FAs as CRB2-wt-HaloTag. Cells in contact show more cortical actin fibers (Figs. 6, *A*, *B* and S8 *F*) and altered FAs. Again, the number of adhesions was lower and on average they were larger; however, the total coverage of FAs was greater than in CRB2 wt-negative AB8/13 cells (Fig. 5, *A* and *B*). Incidentally, CRB2 wt was clearly excluded from the membrane regions of the FAs (Fig. 6, *B* and *D*), which we did not observe in the case for CRB3A wt (Fig. 6, *C* and *E*) and the other examined CRB2 mutants (Fig. S11).

In general, we did not observe an influence of CRB3A wt on the pattern of the FAs. When CRB3A wt was expressed in AB8/13 cells the FAs were similar in size, numbers, and total area compared with CRB2 wt-negative AB8/13 cells (Fig. S8, *B*–*D*). Nevertheless, we detected a certain effect of CRB3A wt on actin fibers. Similar to CRB2 wt-positive cells we also saw an accumulation of cortical actin in cells expressing CRB3A wt (Fig. 6 *C*). However, the extent of peripheral enrichment was not the same in all cells (Fig. 6 *C*), and some CRB3A wt cells in contact showed greater enrichment than others (Fig. S8 *A*). This was also reflected in the quantitative evaluation of the actin intensity profiles (Fig. S8, *E*–*G*). While practically all analyzed CRB2 wt-positive cells showed a clearly increased peripheral actin intensity compared with the CRB2 wt-negative AB8/13 cells (Fig. S8, *F* vs. E), we also found a peripheral actin signal in the cells expressing CRB3A wt (Fig. S8 *G*).

In summary, the traction force microscopy data and the immunolabeling experiments suggest that CRB2 wt acts in *trans* and can recognize neighboring CRB2-expressing cells. This CRB2 wt-CRB2 wt interaction then leads to a massive rearrangement of the cytoskeleton and cell morphology of podocytes in direct contact. It is noteworthy that CRB2 wt does not strengthen the binding of the cells to each other, but ensures a strong substrate anchoring of the CRB2 wt-positive cells in contact by a special restructuring of the FA points. This effect is CRB2 wt specific and—unlike the influence on the actin cytoskeleton—is not observed with cells expressing CRB3A wt.

Expression of CRB2 and CRB3 can induce a reticular cell growth pattern

Next, we were interested in how CRB2 wt might affect cell-cell interactions and podocyte cell migration on a longer spatial and temporal scale. First hints for the impact of CRB2 wt on cell migration came from wound healing assays. Such assays applied to confluent AB8/13 podocyte cell monolayers revealed that the edges of injured cell cultures grew together very slowly if CRB2 wt was expressed (Fig. S9). This suggested that CRB2 wt has a negative effect upon cell migration. To study this in live cell experiments we co-cultured AB8/13 cells expressing CRB2-wt-GFP with AB8/13 cells expressing only intracellular mCherry. The respective cells were mixed in equal numbers and their subsequent growth was monitored with live-cell time-lapse videos over ∼15 h (Fig. 7 *A*).

Once CRB2 wt-positive cells came into direct contact with each other they started establishing extensive cell-cell contacts, which were enriched in CRB2 wt. These cell connections were very stable and existed throughout the measurement for several hours (Fig. 7). The enrichment of CRB2 wt at the cell-cell interface was only observed when two CRB2 wt-positive cells made contact (Video S1). In the contact area between a cell expressing CRB2-wt-GFP and a cell expressing intracellular mCherry, no enrichment of CRB2-wt-GFP at the cell-cell membrane interface was observed (Fig. 7 *A*).

To visualize the effect of the CRB2 wt-dependent interaction directly we investigated co-cultures of cells expressing CRB2 wt either as GFP or as HaloTag fusion protein labeled by JF646 (Fig. 7 *B*; Video S2). Migrating cells first made contact via their splayed microvilli before forming the typical podocyte cell-cell contact interface (Fig. 7 *B*). In Fig. 7 *B*, the CRB2 wt enrichment of the GFP and HaloTag fusion proteins can be seen as distinct yellow areas in the overlaid image.

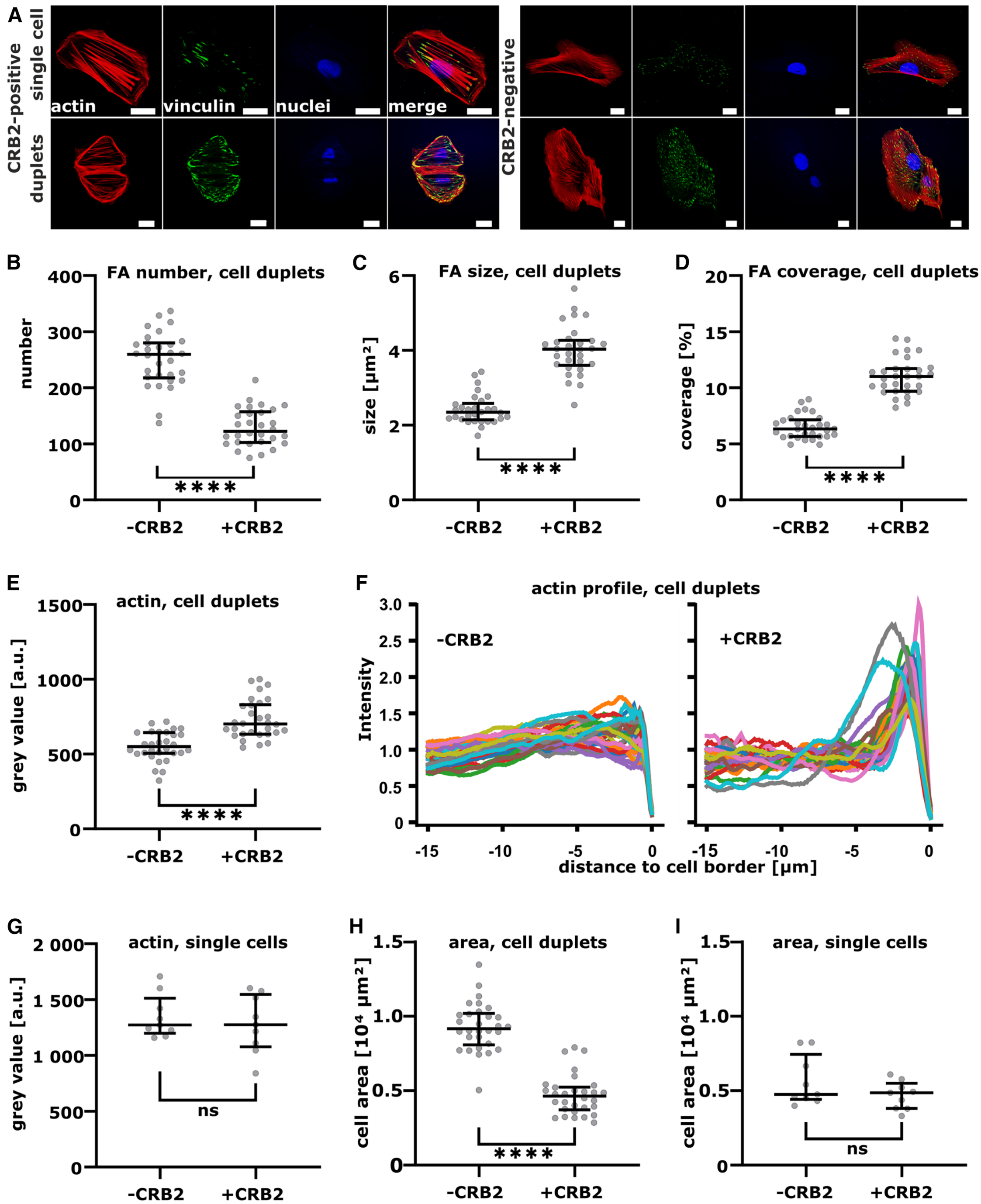

FIGURE 5 Immunofluorescence analyses of AB8/13 podocytes. (*A*) CRB2-positive or CRB2-negative AB8/13 podocytes were fixed and immunocytochemically stained against actin and vinculin. Nuclei were stained with DAPI. Scale bars, 20 μm. Note the different print magnifications. (*B*) Cell duplets, number of FA sites per cell. (*C*) Cell duplets, average FA sizes. (*D*) Cell duplets, fraction of cell area covered by FAs. (*E*) Cell duplets, actin intensity averaged over the whole duplet. (*F*) Cell duplets, actin intensity profiles. (*G*) Single cells, actin intensity. (*H*) Cell duplets, areas of duplets. (*I*) Single cells, area of cell. Sample size, 30 duplets (both conditions) from three independent samples; nine single cells. Shown are individual values, medians and quartiles. Significances were tested by unpaired *t*-test (GraphPad Prism 10, GraphPad; ns, not significant, ****$p < 0.0001$).

From earlier studies on crb2a/crb3a in zebrafish, it was known that crb2a but not crb3a overexpression in HEK cells could induce cell aggregation (21). To examine aggregation behavior for human CRB2 wt and CRB3A wt in podocytes, we co-cultured wt CRB2- or CRB3A-GFP with mCherry-expressing AB8/13 cells. Cells were seeded in low numbers (Fig. S10 *A*) and then the cell mixtures were incubated for 7 days to allow the cells to spread over the entire dish.

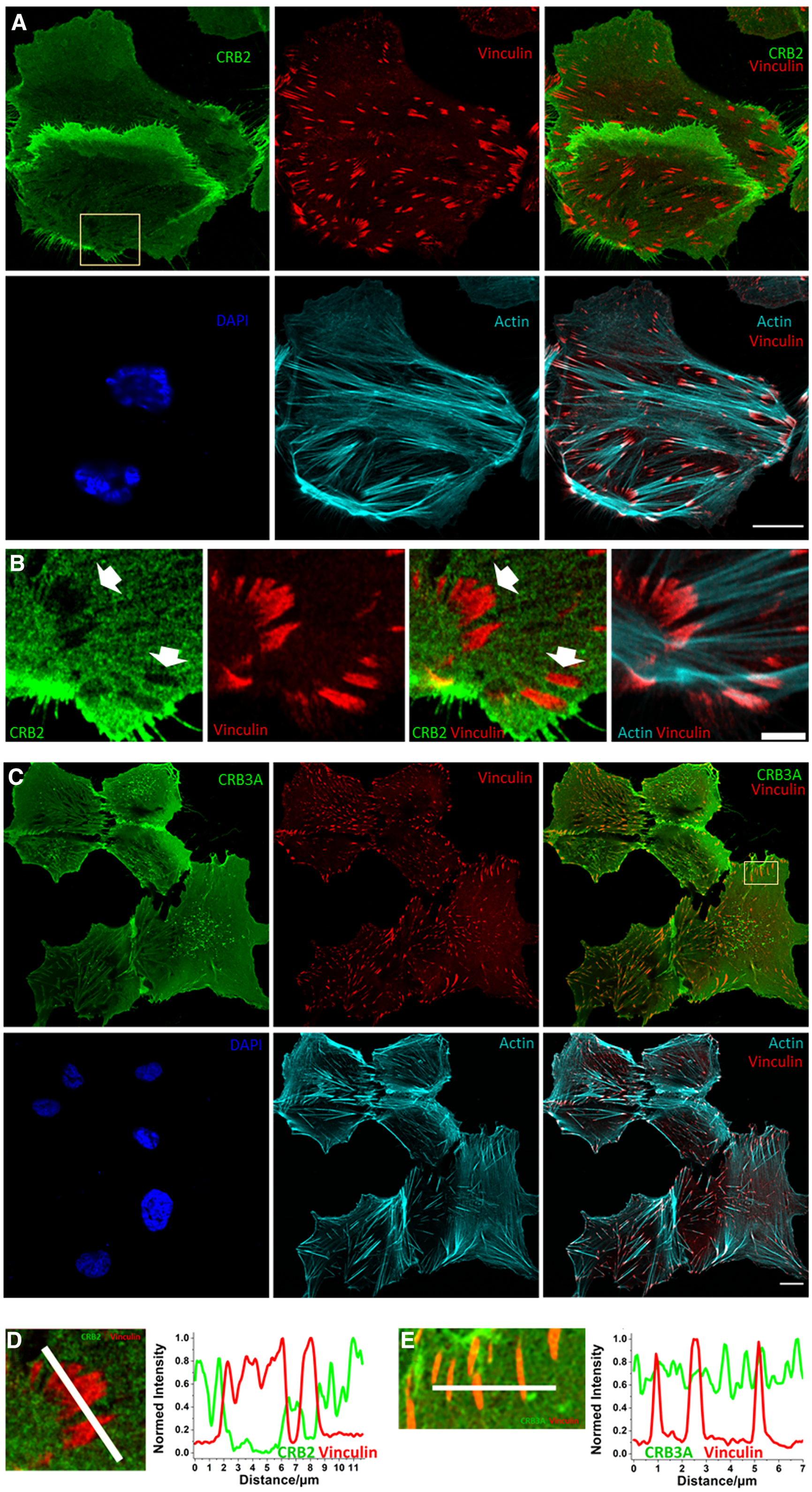

FIGURE 6 Actin and vinculin staining in CRB2-wt-GFP- and CRB3A-wt-GFP-expressing AB8/13 podocytes. (*A*) CRB2-wt-GFP-expressing AB8/13 cells. Scale bar, 20 μm. (*B*) Enlargement of the region marked in (*A*). Scale bar, 5 μm. Vinculin areas that are devoid of CRB2-wt-GFP are marked with arrowheads. (*C*) CRB3A-wt-GFP-expressing AB8/13 cells. Scale bar, 20 μm. (*D*) Line profile of vinculin and CRB2-wt-GFP signal, magnification of the yellow box in (*A*). (*E*) Line profile of vinculin and CRB3A-wt-GFP, magnification of the yellow box in (*C*).

Similarly, mCherry-expressing AB8/13 cells were grown together with CRB3ΔICD-expressing AB8/13 cells as control. The distribution of the respective cell types was then analyzed after fixation and nuclear staining of the cells. The entire cell culture dish was imaged with tile scans, which were later stitched into a single image (Fig. 8 *A*).

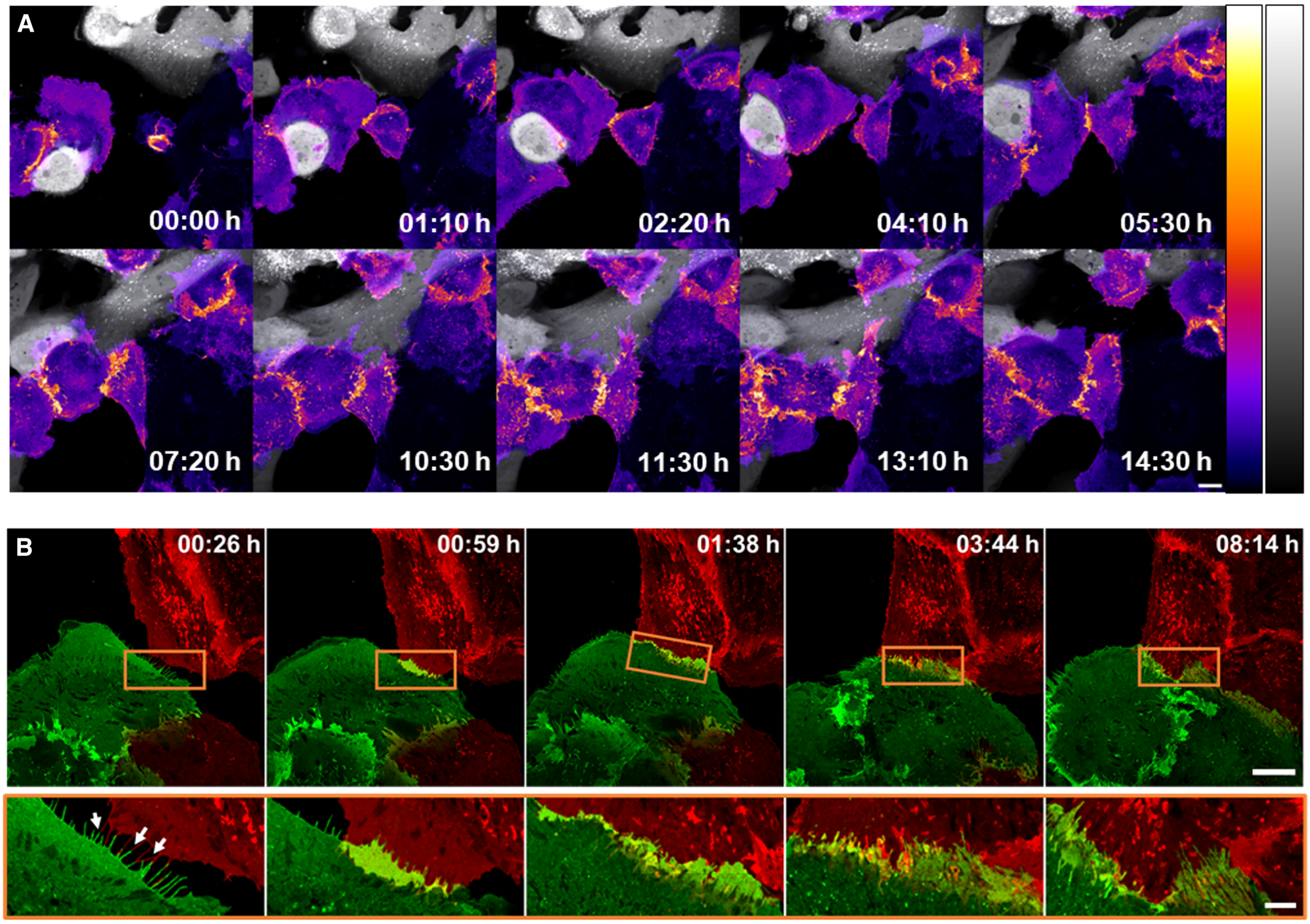

FIGURE 7 Time lapse live-cell images of co-cultured AB8/13 cells. (*A*) AB8/13 podocytes expressing either intracellular mCherry (*grayscale lookup table*) or CRB2 wt fused to GFP (*fire color lookup table*) were co-cultivated. Equal numbers of both cell types were seeded together in a glass-bottom cell culture dish and imaged for ∼15 h. Confocal images were taken every 10 min (Video S1). Scale bar, 10 μm. (*B*) AB8/13 podocytes expressing CRB2 wt either fused to HaloTag labeled by JF646 (*red*) or GFP (*green*) were co-cultivated. Equal numbers of both cell types were seeded together in a glass-bottom cell culture dish and imaged for ∼9 h. Confocal images were taken every 7 min (Video S2). Top row shows the full image. Scale bar, 25 μm. Bottom row, the magnified area from the orange rectangle. Scale bar, 5 μm.

Figure360▷ For a Figure360 author presentation of Figure 7, see https://doi.org/10.1016/j.bpj.2025.07.020#mmc7.

These images alone revealed interesting and distinct growth patterns for CRB2-wt-GFP-positive and CRB2 wt-negative cells. To quantify these patterns the images from the GFP channel were translated into a binary mask to allow for identification of contiguous clusters of CRB2 wt-expressing cells by their perimeter using respective image analysis (see scheme in Fig. 8 *B*). CRB3ΔICD cells (Fig. 8 *D*, *right column*) served as a negative control and the perimeter of the biggest cluster found in this dish was used as threshold to sort out “random cell clusters” in the CRB2 wt (Fig. 8 *D*, *left column*) and CRB3A wt cell populations (Fig. 8 *D*, *middle column*). The results indicated that CRB2 wt and also CRB3A wt expression induced a specific growth pattern with cells arranged in extended and interconnected cell clusters resulting in a long-range reticular meshwork (Fig. 8 *D*, see also Videos S3, S4, and S5). This behavior was reproduced in two more independent experiments (Fig. S10, *B* and *C*). We quantified the patterning effect from the three independent sets of experiments by counting all cells using their nuclear DNA staining and plotting the number of cells in CRB2 wt or CRB3A wt clusters normalized to all cells in the dish (Fig. 8, *B* and *C*). CRB2 wt-expressing cells clustered significantly more than the CRB3ΔICD control cells ($p < 0.05$).

DISCUSSION

In this study, we aim to understand the exact role of the mammalian homologs CRB2 and CRB3A in human podocytes. To date, much is known about the function of CRB3A; therefore, we focused on CRB2 in relation to CRB3A. To this end we used AB8/13 cells as simplified 2D-cell culture model, which is well established to mimic human podocyte behavior in vitro. In these AB8/13 cells, we expressed CRB2 wt, CRB3A wt, and several mutants of these, as well as fusions with GFP and HaloTag.

First of all, we found that CRB2 has a particularly anisotropic distribution at the cell-cell contact surfaces compared

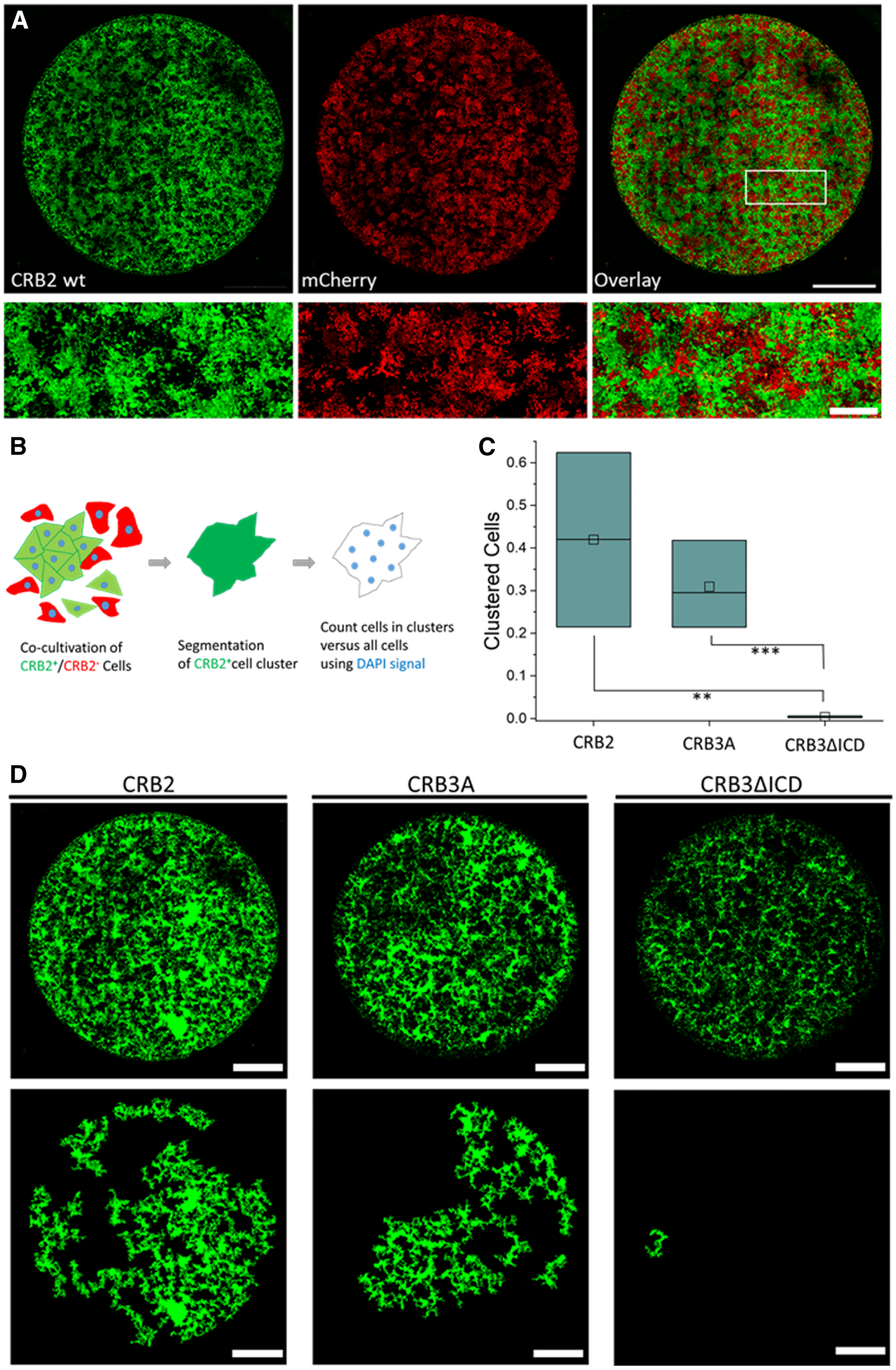

FIGURE 8 Growth pattern analysis of co-cultivated AB8/13 podocytes. (*A*) Same numbers of AB8/13 podocytes expressing either CRB2 wt, CRB3Awt, or CRB3ΔICD fused to GFP were seeded together with cells expressing only mCherry on a plate, then fixed and imaged after 7 days. Scale bars, 5 and 1 mm. The lower row shows enlargements of the regions marked above. (*B*) The GFP signal was used for image segmentation and identification of CRB2 wt-expressing cell clusters. (*C*) Ratio of cells in clusters and all cells based on the cluster segmentation and DAPI staining of the nuclei (***$p < 0.01$, **$p < 0.05$). (*D*) Top row: binary images of the GFP channel. Bottom row: binary image after subtraction of clusters smaller than the maximum random cluster size. Scale bar, 5 mm.

with CRB3A. Therefore, we investigated CRB2 membrane dynamics and, in particular, CRB2 behavior at the cell-cell membrane contacts using FRAP. The results of the experiments suggested a highly dynamic setting at the podocyte-podocyte membrane interface, where CRB2 interacted in *trans* and was partly immobilized when podocytes came into direct contact. Surprisingly, upon removal of CRB2's ECD, we observed an increase of the CRB2 immobilization in this membrane region such that almost 40% of the CRB2ΔECD molecules were immobilized, in single measurements even up to 85%. This suggested that intracellular interactions at the cell-cell membrane interface arrested CRB2 and that a functional extracellular CRB2 domain was required to trigger its release. This trigger could eventually arise from the direct *trans* interaction of two CRB2 ECDs.

Our computational molecular structure had indicated that CRB2 is a flexible molecule with three hinge-like domains in its ECD. Thus, presumably the extracellular part of the CRB2 molecule is remodeled upon CRB2-CRB2 interactions, and this structural change represents the signal for the intracellular release of the ICD domain and triggers a subsequent cellular response (Fig. 9).

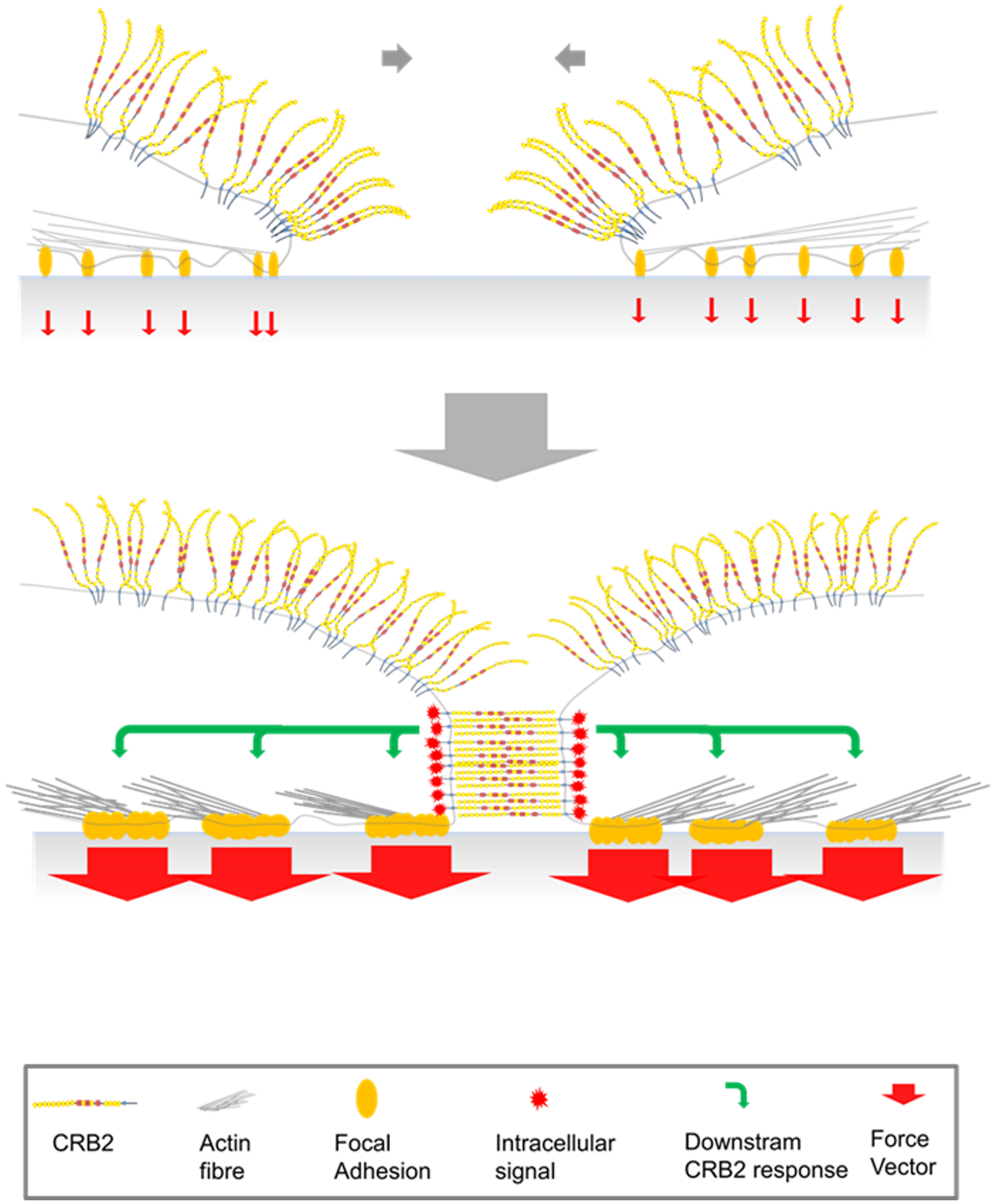

FIGURE 9 Model of CRB2-dependent podocyte cell-matrix anchorage. CRB2-CRB2 *trans* interactions at the cell-cell membrane interface trigger increased cell-matrix adhesion by increasing the FA size and their overall cell coverage when two CRB2-expressing podocytes come into close contact (*green arrows*). Consequently, the force (*red arrows*) onto the cell matrix exerted by the podocyte is significantly stronger after the CRB2-induced cellular response.

The CRB2ΔECD retardation was only observed at the cell-cell membrane interface of podocytes in direct contact. In other—free—plasma membrane regions, removal of the ECD from CRB2 showed an opposite effect and resulted in a fast and CRB3A-like mobility pattern. CRB3A was generally diffusing faster than CRB2 within the podocyte membrane and also did not show a notable delay at the cell-cell membrane interface and no notable increase of its immobile fraction. CRB3A displayed a lateral membrane mobility that was only determined by its transmembrane domain since deletion of its ICD did not increase its mobility. This was particularly remarkable considering how similar are the ICD's of CRB3A and CRB2. However, previous in vitro binding experiments with PALS1 have

shown that the binding constant of the CRB1 ICD, which is identical to the CRB2 ICD, is roughly seven times stronger than the respective CRB3A binding constant (23). Differences in the binding strength to such intracellular interaction partners might well explain that CRB2—depending on its ECD status—can be immobilized, while CRB3A cannot. Interestingly, deletion or functional inactivation of the intracellular PDZ and FERM binding motifs of CRB2 did not exert any significant effect on mobility, neither in the free plasma membrane nor the membrane interfaces of neighbored podocytes. Of course, this does not mean that inactivation of the PBM or FBM has no influence, but only that the associated effects do not manifest themselves in an altered membrane mobility of the respective mutated protein.

Our FRAP data suggested a homophilic CRB2 interaction in *trans*. However, we sought more direct evidence and turned to traction force microscopy of CRB2-expressing cells. We also wanted to know how CRB2 affected the cell-cell connection directly. Our initial hypothesis was that *trans* interactions between CRB2 proteins of adjacent cells would directly stabilize and enhance their cell-cell contacts. This should result in increased attractive forces between two contacting cells. To our surprise, this was not the case. Instead, the contacting CRB2-expressing cells applied higher forces toward the substrate. Our immunochemical analysis of the cellular FAs and the cytoskeleton revealed that this was driven by an enhancement of cellular FA contacts. Thus, CRB2 clearly acts in *trans* but does not directly stabilize the cell-cell contacts. Rather, it acts as a dynamic sensor of the cellular environment, which triggers a very stable cell-matrix anchorage of CRB2-positive podocytes after they have established contact. This is also the reason for the observed slowdown in the migration of podocyte cells as revealed by wound healing assays. Importantly, the effect on FAs was CRB2 specific and did not occur in CRB3A-expressing podocytes.

The results of the long-term migration behavior of CRB2-expressing podocytes were consistent with these results. After 1 week of incubation, the cells formed stable net-like cell clusters that extended over an entire cell culture dish. Our data confirmed and extended older observations from Zhou et al. (21), which expressed zebrafish crb2a and crb2b in HEK cells and showed that they started to grow in clusters. However, the cell clusters they observed were much smaller in terms of number (9–16) and did not expand to the extensive network we observed after expression of human CRB2 in AB8/13 cells comprising in several cases up to ∼200,000 cells. Zhou et al. (21) also observed a *trans* interaction of the extracellular crb2a domain but it was not dynamic or transient but biochemically very stable. Interestingly, they also expressed the untagged zebrafish crb3a in HEK cells but unlike crb2 they did not observe any clustering. Therefore, we were surprised to also observe cell clustering, albeit less pronounced than with CRB2, when we constitutively expressed human CRB3A as GFP fusion proteins in AB8/13 cells. This could not be related to *trans* interactions of the ECD, because CRB3A does not have an extended ECD like CRB2. However, this could be linked to the increased peripheral actin reorganization that we also observed after CRB3A expression in AB8/13 cells. A similar effect has been shown earlier when CRB3A was overexpressed in HeLa cells, which were devoid of endogenous CRB3A (39). HeLa cells normally show a fragmented F-actin distribution, whereas the CRB3A-expressing cells showed a more circumferential actomyosin belt. Interestingly, this reorganization of the cytoskeleton led to an epithelial cell-like morphology of the HeLa cells and allowed them to grow in tightly packed, roundish cell clusters (39).

Crumbs2 is essential for building up the SD at the renal filtration barrier. Recent research from us and other groups using podocyte-specific CRB2 knockout mice demonstrates how the loss of CRB2 impacts the glomerular ultrastructure (14,40). In these mice, loss of Crumbs2 resulted in heavy proteinuria, which was accompanied by progressive podocyte FP effacement (14,40). Interestingly, this CRB2-specific phenotype was very similar to the phenotype of podocyte-specific knockout of vinculin (41). Vinculin connects actin filaments to integrin-mediated cell-matrix adhesions and to cadherin-based intercellular junctions, and both are important for the maintenance of the glomerular filtration barrier (41). Vinculin loss in podocyte-specific vinculin knockout mice also resulted in increased albuminuria and FP effacement following injury in vivo (41). Interestingly, in primary podocytes isolated from these knockout mice, the size of the FAs was altered and cells clearly showed increased cell migration.

Our results are in good agreement with these findings from the CRB2/vinculin knockout experiments and might explain how CRB2 acts in finally building up the SD (Fig. 9). CRB2 is expressed very early in CRB3A-positive renal progenitor cells, long before these cells differentiate into the final glomerular filtration barrier-forming podocytes (10). CRB2, eventually synergistically with CRB3A, might enforce initial clustering of the developing podocyte progenitors. Then, the CRB2-induced tight cell adhesion and very stable cell-matrix anchorage finally allows the mature postmitotic podocytes to build a stable filtration barrier between them. This also explains the pathological effect of CRB2 loss. In the absence of CRB2-induced tight cell-matrix anchorage, the podocytes can detach easily under stress conditions leading to the observed detachment of the FPs and the breakdown of the filtration barrier. This is also supported by in vitro cell adhesion assays where human CRB2 knockout podocytes show decreased adhesive ability (40).

The results of our study expand our understanding of podocyte biology and the pathogenicity of CRB2 loss. Furthermore, a new general aspect on the interaction of

the ECDs of CRB proteins in specific biological contexts is added. There is a *trans* interaction that acts as a dynamic homophilic cell sensor rather than a stable protein-protein connection. However, one must keep in mind that our results are derived from a very simplistic cell culture model of the otherwise extremely complex podocyte cell architecture. A 2D cell culture model can of course never fully mimic the sophisticated 3D arrangement of podocytes in their glomerular environment in situ. The precise interaction within the glomeruli of their FPs is therefore not really reproducible either, not to mention the apicobasal polarity, which is often important in the context of CRB biology. Further experiments are needed to confirm the generality of our results. However, it would be extremely exciting, for example, to test known and clinically relevant point mutants of the CRB2 ECD to see whether their pathogeny relates to their function as an extracellular sensor being either disrupted or even misdirected.

DATA AND CODE AVAILABILITY

All data are available in the main text or the supporting material. All microscopic raw data can be made obtainable for download after request to the correspondent author.

ACKNOWLEDGMENTS

This publication is dedicated to the memory of Erich Sackmann who taught R.M. how to be a scientist. Erich's groundbreaking work on mechanobiology of cells and his rigorous physics-based approach is an ongoing inspiration to all authors. We thank Sabine Wirths for her excellent technical assistance. We also acknowledge molecular graphics and analyses performed with UCSF ChimeraX, developed by the Resource for Biocomputing, Visualization, and Informatics at the University of California, San Francisco, with support from National Institutes of Health R01-GM129325 and the Office of Cyber Infrastructure and Computational Biology, National Institute of Allergy and Infectious Diseases. This work is part of the PhD thesis of R.B. (42). This study was funded by the German Research Foundation grant KU 2474/12-2 (to U.K.), WE 2550/5-1 and WE 2550/2-2 (to T.W.), and 363055819/GRK2415 (to R.M.).

AUTHOR CONTRIBUTIONS

Conceptualization, J.P.S., T.W., and U.K.; methodology, J.P.S., U.K., R.B., and B.H.; investigation, R.B., A.M.-K., S.G., Y.H., R.H., and R.R.; visualization, R.B., A.M.-K., and S.G.; supervision, J.P.S., U.K., T.W., and R.M.; writing – original draft, J.P.S. and R.B.; writing – review & editing, J.P.S., T. W., U.K., and R.M.; funding acquisition, T.W., U.K., and R.M.

DECLARATION OF INTERESTS

The authors declare no competing interests.

SUPPORTING MATERIAL

Supporting material can be found online at https://doi.org/10.1016/j.bpj.2025.07.020.

REFERENCES

1. Tan, B., S. M. J. M. Yatim, …, A. Ludwig. 2020. The Mammalian Crumbs Complex Defines a Distinct Polarity Domain Apical of Epithelial Tight Junctions. *Curr. Biol.* 30:2791–2804.e6.
2. Thompson, B. J., F. Pichaud, and K. Röper. 2013. Sticking together the Crumbs - an unexpected function for an old friend. *Nat. Rev. Mol. Cell Biol.* 14:307–314.
3. Rodriguez-Boulan, E., and I. G. Macara. 2014. Organization and execution of the epithelial polarity programme. *Nat. Rev. Mol. Cell Biol.* 15:225–242.
4. Quinn, P. M., L. P. Pellissier, and J. Wijnholds. 2017. The CRB1 Complex: Following the Trail of Crumbs to a Feasible Gene Therapy Strategy. *Front. Neurosci.* 11:175.
5. Xiao, Z., J. Patrakka, …, K. Tryggvason. 2011. Deficiency in Crumbs homolog 2 (Crb2) affects gastrulation and results in embryonic lethality in mice. *Dev. Dyn.* 240:2646–2656.
6. Ramkumar, N., T. Omelchenko, …, K. V. Anderson. 2016. Crumbs2 promotes cell ingression during the epithelial-to-mesenchymal transition at gastrulation. *Nat. Cell Biol.* 18:1281–1291.
7. Alves, C. H., K. Bossers, …, J. Wijnholds. 2013. Microarray and morphological analysis of early postnatal CRB2 mutant retinas on a pure C57BL/6J genetic background. *PLoS One.* 8:e82532.
8. Tait, C. M., K. Chinnaiya, …, M. Placzek. 2020. Crumbs2 mediates ventricular layer remodelling to form the spinal cord central canal. *PLoS Biol.* 18:e3000470.
9. Dudok, J. J., M. Murtaza, …, J. Wijnholds. 2016. Crumbs 2 prevents cortical abnormalities in mouse dorsal telencephalon. *Neurosci. Res.* 108:12–23.
10. Hamano, S., Y. Nishibori, …, K. Yan. 2018. Association of crumbs homolog-2 with mTORC1 in developing podocyte. *PLoS One.* 13:e0202400.
11. Hochapfel, F., L. Denk, …, M. P. Krahn. 2017. Distinct functions of Crumbs regulating slit diaphragms and endocytosis in Drosophila nephrocytes. *Cell. Mol. Life Sci.* 74:4573–4586.
12. Ebarasi, L., S. Ashraf, …, A. Majumdar. 2015. Defects of CRB2 cause steroid-resistant nephrotic syndrome. *Am. J. Hum. Genet.* 96:153–161.
13. Slavotinek, A., J. Kaylor, …, L. Mehta. 2015. CRB2 mutations produce a phenotype resembling congenital nephrosis, Finnish type, with cerebral ventriculomegaly and raised alpha-fetoprotein. *Am. J. Hum. Genet.* 96:162–169.
14. Moller-Kerutt, A.…, 2021. Crumbs2 Is an Essential Slit Diaphragm Protein of the Renal Filtration Barrier. *J. Am. Soc. Nephrol.* 32:1053–1070.
15. Quinn, P. M., T. M. Buck, …, J. Wijnholds. 2019. Human iPSC-Derived Retinas Recapitulate the Fetal CRB1 CRB2 Complex Formation and Demonstrate that Photoreceptors and Muller Glia Are Targets of AAV5. *Stem Cell Rep.* 12:906–919.
16. Charrier, L. E., E. Loie, and P. Laprise. 2015. Mouse Crumbs3 sustains epithelial tissue morphogenesis in vivo. *Sci. Rep.* 5:17699.
17. Whiteman, E. L., S. Fan, …, B. Margolis. 2014. Crumbs3 is essential for proper epithelial development and viability. *Mol. Cell Biol.* 34:43–56.
18. Grahammer, F., C. Schell, and T. B. Huber. 2013. The podocyte slit diaphragm–from a thin grey line to a complex signalling hub. *Nat. Rev. Nephrol.* 9:587–598.
19. Scott, R. P., and S. E. Quaggin. 2015. Review series: The cell biology of renal filtration. *J. Cell Biol.* 209:199–210.
20. Kopp, J. B., H. J. Anders, …, P. Romagnani. 2020. Podocytopathies. *Nat. Rev. Dis. Primers.* 6:68.
21. Zou, J., X. Wang, and X. Wei. 2012. Crb apical polarity proteins maintain zebrafish retinal cone mosaics via intercellular binding of their extracellular domains. *Dev. Cell.* 22:1261–1274.
22. Saleem, M. A., M. J. O'Hare, …, P. Mundel. 2002. A conditionally immortalized human podocyte cell line demonstrating nephrin and podocin expression. *J. Am. Soc. Nephrol.* 13:630–638.

23. Li, Y., Z. Wei, …, M. Zhang. 2014. Structure of Crumbs tail in complex with the PALS1 PDZ-SH3-GK tandem reveals a highly specific assembly mechanism for the apical Crumbs complex. *Proc. Natl. Acad. Sci. USA.* 111:17444–17449.

24. Schindelin, J., I. Arganda-Carreras, …, A. Cardona. 2012. Fiji: an open-source platform for biological-image analysis. *Nat. Methods.* 9:676–682.

25. Hersch, N., B. Wolters, …, B. Hoffmann. 2013. The constant beat: cardiomyocytes adapt their forces by equal contraction upon environmental stiffening. *Biol. Open.* 2:351–361.

26. Ahrens, D., W. Rubner, …, R. Merkel. 2019. A Combined AFM and Lateral Stretch Device Enables Microindentation Analyses of Living Cells at High Strains. *Methods Protoc.* 2:43.

27. Merkel, R., N. Kirchgessner, …, B. Hoffmann. 2007. Cell force microscopy on elastic layers of finite thickness. *Biophys. J.* 93:3314–3323.

28. Houben, S., N. Kirchgeßner, and R. Merkel. 2010. Estimating Force Fields of Living Cells - Comparison of Several Regularization Schemes Combined with Automatic Parameter Choice. *In* BerlinPattern Recognition.Proceedings of the DAGM Symposium, Lecture Notes in Computer Sciences 6376. M. Goesele, S. Roth, A. Kuijper, B. Schiele, and K. Schindler, eds Springer.

29. Butler, J. P., I. M. Tolić-Nørrelykke, …, J. J. Fredberg. 2002. Traction fields, moments, and strain energy that cells exert on their surroundings. *Am. J. Physiol. Cell Physiol.* 282:C595–C605.

30. OriginPro, Version 2021, in OriginLab Corporation. Northampton, MA, USA.

31. Almagro Armenteros, J. J., K. D. Tsirigos, …, H. Nielsen. 2019. SignalP 5.0 improves signal peptide predictions using deep neural networks. *Nat. Biotechnol.* 37:420–423.

32. Baek, M., F. DiMaio, …, D. Baker. 2021. Accurate prediction of protein structures and interactions using a three-track neural network. *Science.* 373:871–876.

33. Meng, E. C., T. D. Goddard, …, T. E. Ferrin. 2023. UCSF ChimeraX: Tools for structure building and analysis. *Protein Sci.* 32:e4792.

34. Lippincott-Schwartz, J., E. L. Snapp, and R. D. Phair. 2018. The Development and Enhancement of FRAP as a Key Tool for Investigating Protein Dynamics. *Biophys. J.* 115:1146–1155.

35. Zeiss, ZEN Microscopy Software Zeiss: https://www.zeiss.com/microscopy/en/products/software/zeiss-zen.html.

36. Djuric, I., J. P. Siebrasse, …, T. Weide. 2016. The C-terminal domain controls the mobility of Crumbs 3 isoforms. *Biochim. Biophys. Acta.* 1863:1208–1217.

37. Dembo, M., and Y. L. Wang. 1999. Stresses at the cell-to-substrate interface during locomotion of fibroblasts. *Biophys. J.* 76:2307–2316.

38. Cesa, C. M., N. Kirchgessner, …, R. Merkel. 2007. Micropatterned silicone elastomer substrates for high resolution analysis of cellular force patterns. *Rev. Sci. Instrum.* 78:034301.

39. Loie, E., L. E. Charrier, …, P. Laprise. 2015. CRB3A Controls the Morphology and Cohesion of Cancer Cells through Ehm2/p114RhoGEF-Dependent Signaling. *Mol. Cell Biol.* 35:3423–3435.

40. Tanoue, A., K. Katayama, …, K. Dohi. 2021. Podocyte-specific Crb2 knockout mice develop focal segmental glomerulosclerosis. *Sci. Rep.* 11:20556.

41. Lausecker, F., X. Tian, …, S. Ishibe. 2018. Vinculin is required to maintain glomerular barrier integrity. *Kidney Int.* 93:643–655.

42. Bhatia, R. 2024. *In* Relevance of Crumbs homolog 2 in the Glomerular filtration barrier. Clausius-Institut für Physikalische und Theoretische Chemie Rheinische Friedrich-Wilhelms-Universität Bonn https://bonndoc.ulb.uni-bonn.de/xmlui/handle/20.500.11811/12590.

Biophysical Journal, Volume *125*

Supplemental information

Crumbs2 acts as a dynamic cellular sensor triggering stable podocyte cell-matrix anchorage

Rohan Bhatia, Annika Möller-Kerutt, Sven Gerlach, Yannick Herfs, Rebecca Rixen, Hermann Pavenstädt, Bernd Hoffmann, Rudolf Merkel, Ulrich Kubitscheck, Thomas Weide, and Jan Peter Siebrasse

Biophysical Journal

Supplemental Materials

This PDF file includes:

Figs. S1 to S11

Other Supplemental Materials for this manuscript include the following:

Movies S1 to S5

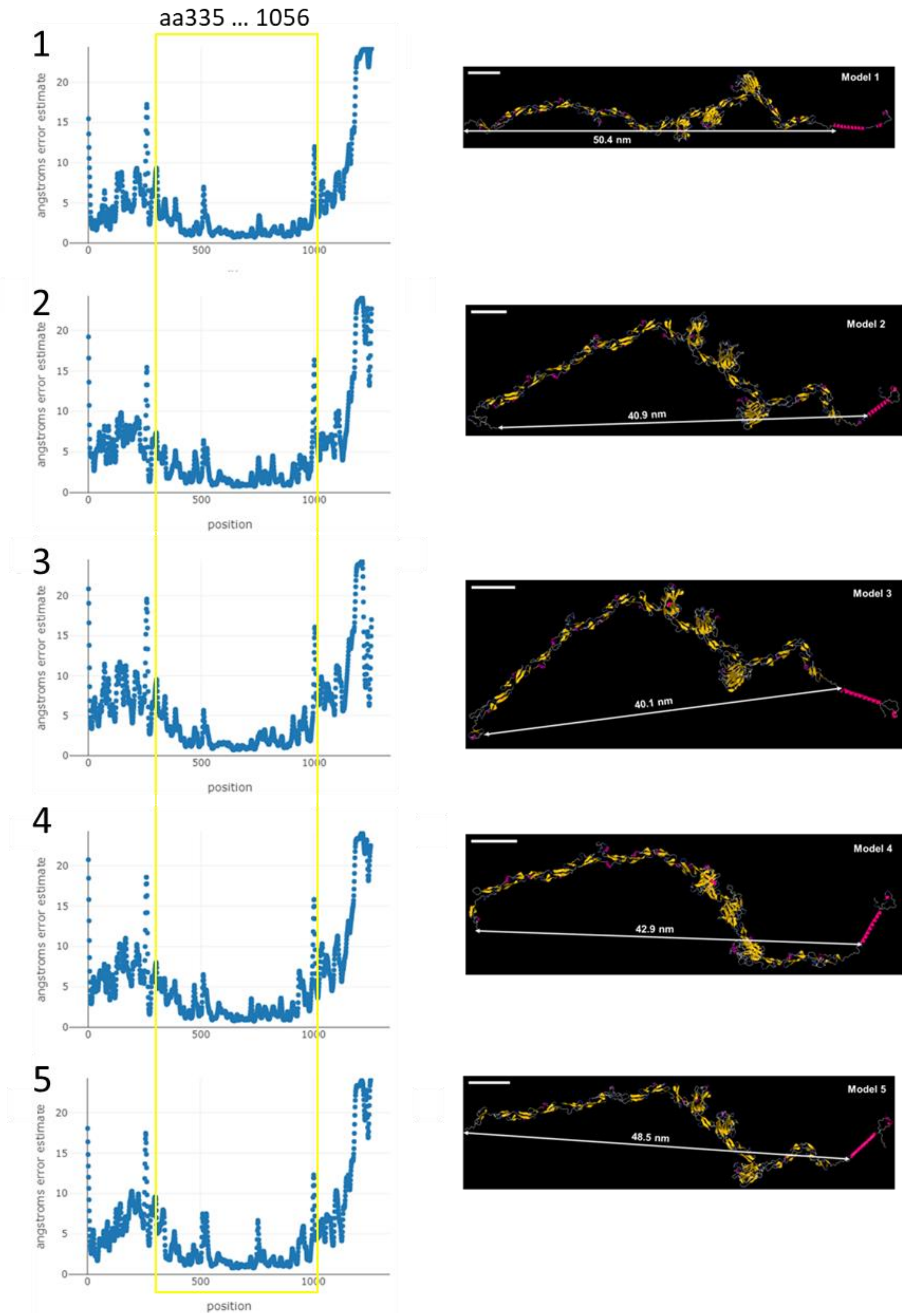

Fig. S1: In-silico prediction of the CRB2 protein structure.
Five different computational models of CRB2 wt computed with the RosettaFold software [23] (right column) with their respective error estimate (left column).

Western blots

Western blots were prepared to compare the expression level of constitutively or inducibly controlled GFP tagged variants of CRBs proteins against α-tubulin which was chosen as the housekeeping gene. All CRB variants showed typical double bands at the expected sizes (CRB2 wt, CRB2ΔFBM and CRB2ΔPBM: ~180-200 kDa.; CRB2ΔECD : ~70 kDa.; CRB3A wt: ~55 kDa. and CRB3ΔICD: < 55 kDa.) indicating proper glycosylation and transfer to the PM of these CRB proteins in all cell lines as shown in Figure 2 and Figure S11. An additional, non-specific band at approximately 130 kDa. was also visible, likely due to antibody cross-reactivity as it appeared also for the control cell line (represented as * in Figure S2).
Comparing the expression level for the constitutively expressing cells (Figure S2A), CRB 3A and 3ΔICD were clearly similarly expressed as the housekeeping gene whereas CRB2 was not. On the other hand, in the case of the inducible promoter (Figure S2B), all CRBs variants showed lower expression as compared to the housekeeping gene. Weaker α-tubulin bands were visualized for the constitutive CRB3ΔICD and inducible CRB2ΔECD owing to lower cell counts at harvest.

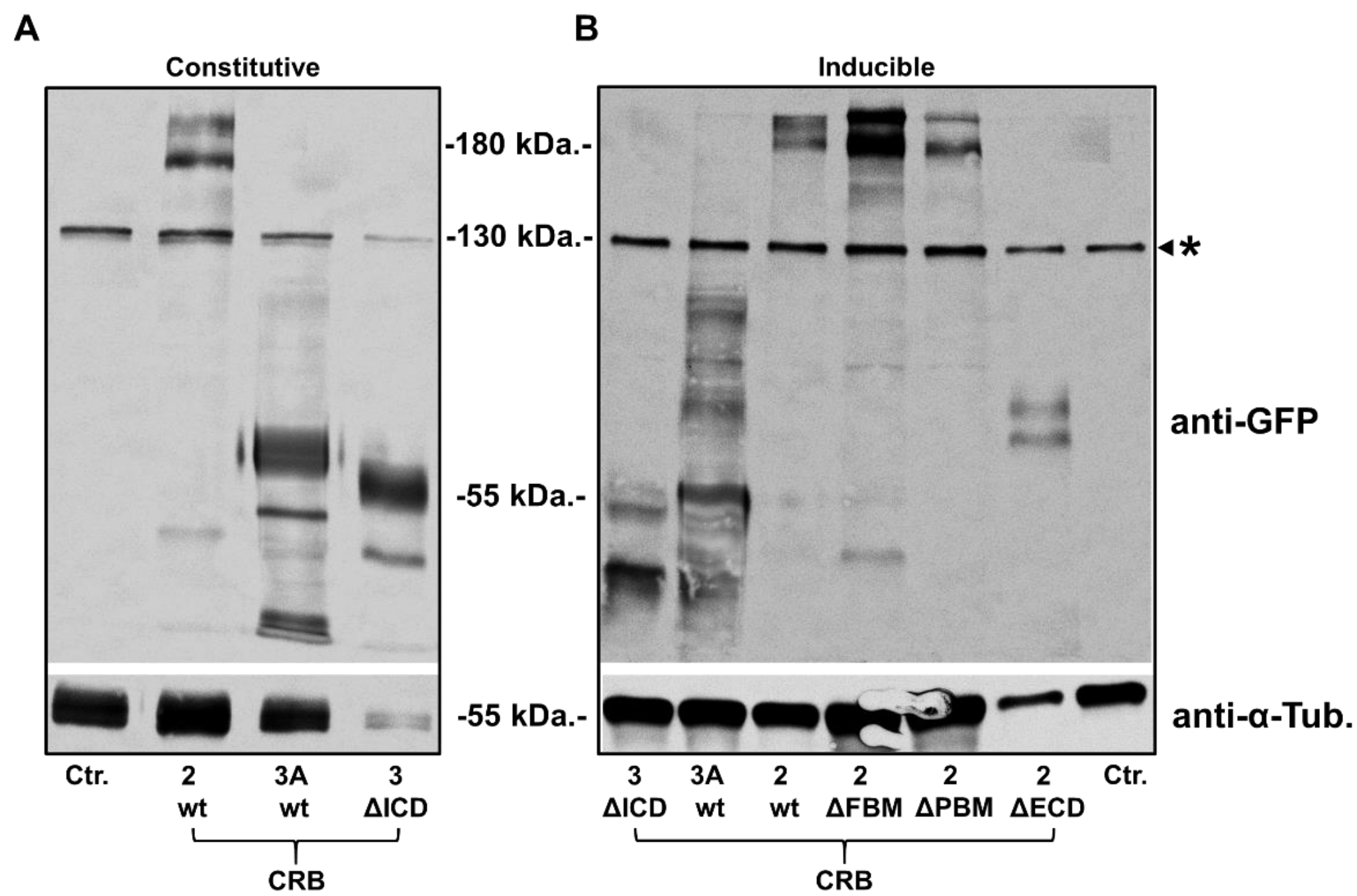

Figure S2: Western Blot analysis of AB8 cells.
(A) Constitutively expressing GFP tagged versions of CRB3ΔICD, CRB3A wt and CRB2 wt along with a cell line not expressing any GFP tagged protein (Ctr.). (B) Inducible GFP tagged versions of CRB3ΔICD, CRB3A wt, CRB2 wt, CRB2ΔFBM, CRB2ΔPBM and CRB2ΔECD along with a cell line not expressing any GFP tagged protein (Ctr.). Induction was initiated by treatment with 200 mM doxycycline for 48 hours. All tested CRB variants show the characteristic double band at the expected sizes. CRB2 wt, CRB2ΔFBM and CRB2ΔPBM: ~180-200 kDa.; CRB2ΔECD: ~70 kDa.; CRB3A wt: ~55 kDa. and CRB3ΔICD: < 55 kDa., which suggests proper glycosylation and folding of the protein. (*) represents the observed cross activity from the GFP antibody at ~130 kDa.

FRAP_measurements

For FRAP measurements three different region of interest (ROI) with diameters of 2 µm were defined. ROI1 served as bleach mask, ROI2 was used to detect bleaching during image recoding and ROI3 provided the background signal (see Fig. S2A and C). In ROI1, GFP fluorescence was bleached by 5 repeated scans with 100% transmission of the argon (488nm and 514nm) and UV lasers (405nm) (Fig. S2B and D). When the bleach ROI was placed in the brighter contact region of two adjacent cells like in Fig. S2A the recording was categorized as "cell-cell membrane interface" and when the bleach ROI was located somewhere within the cell away from the cell-cell contacts like in Fig. S2C it was categorized as "free plasma membrane". For the free plasma membrane recordings we avoided the ER and the direct nuclear region or any visible intracellular vesicles (Fig. S2 C). Five pre-bleach images were taken and the fluorescence recovery was then recorded for 120 s after bleaching. Raw data for a single measurement were shown in Fig. S2E. The bleach and background corrected curves of 90 measurements were then averaged (Fig. S2F). However, time constants and maximum recovery was calculated from single curves (Fig. S2F; blue triangles).

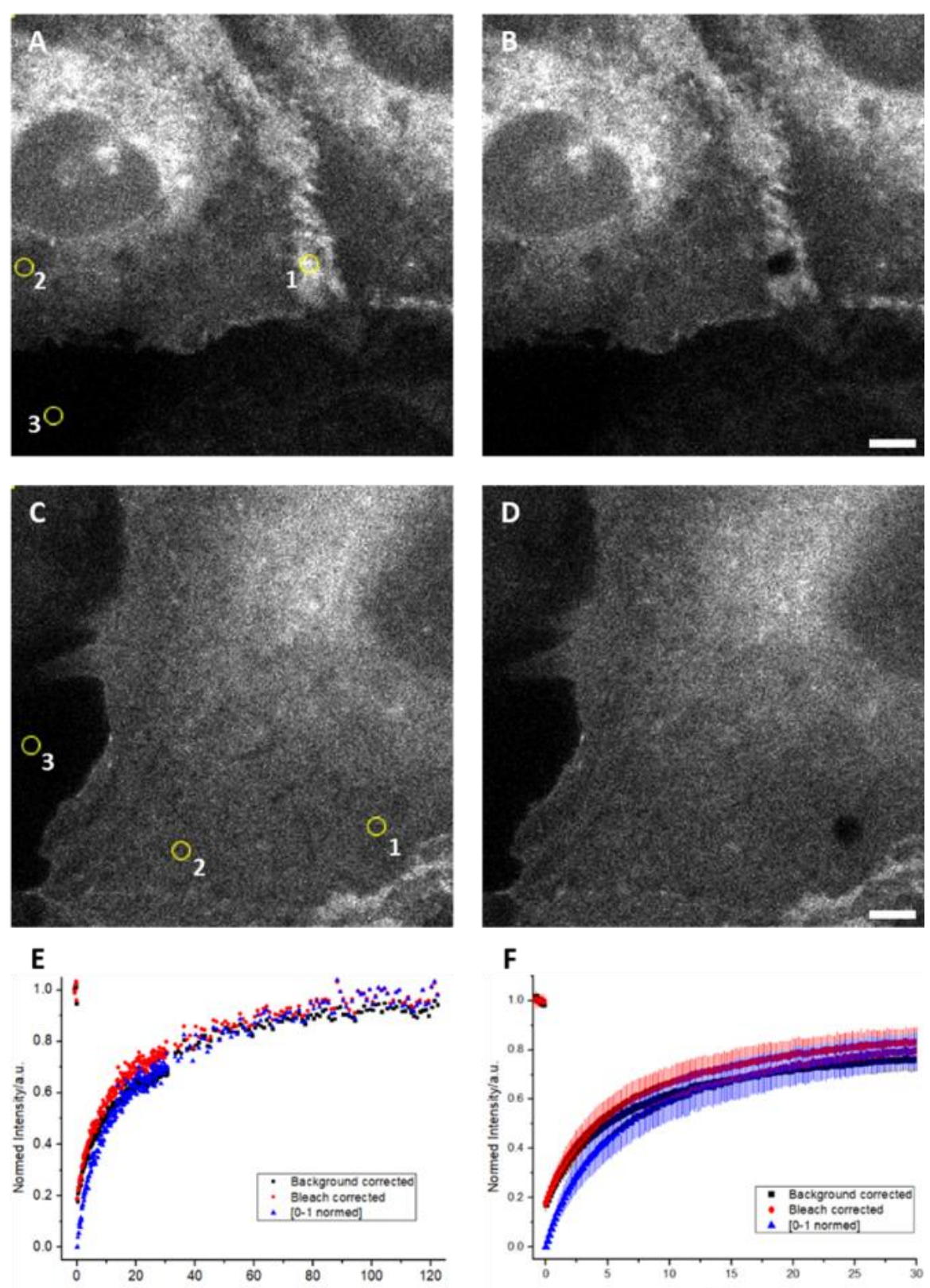

Fig. S3: FRAP Measurements of AB8/13 podocytes expressing CRB2 wt. Scale bar 5 µm
(A) Placement of the three ROI's for measuring the mobility at the membrane-membrane contact interface. Position 1 yielded the signal of the ROI in question, $F_1(t)$. To monitor the bleaching during recording a second ROI was placed somewhere else in the membrane yielding $F_2(t)$. For background correction we used a third ROI outside the cell (3) yielding F_3. From the three intensity values we calculated a normalized recovery signal as a function of time according to:

$$f(t) = \frac{(F_1(t) - F_3) \cdot (F_2(0) - F_3)}{(F_1(0) - F_3) \cdot (F_2(t) - F_3)}$$

Equation 1

$F_1(0)$ and $F_2(0)$ correspond to the prebleach intensity values. (B) First image after bleaching the cell shown in (A). (C) Placement of the three ROIs for measuring the mobility in the free plasma membrane. (D) First image after bleaching the cell shown in (C). (E) Raw data for a single FRAP measurement. (F) Averaged data curve from 90 measurements recorded from three independent experiments. The vertical lines display the standard deviation of the data at the individual time points. To illustrate the rapid recovery, only the first 30 seconds of the acquired data are shown. Both the individual FRAP curves and the averaged curves were parameterized by the following double-exponential growth curve:

$$f(t) = A_1 \cdot (1 - e^{-t/\tau_1}) + A_2 \cdot (1 - e^{-t/\tau_2})$$

Equation 2

The time constant τ_1 was interpreted as a measure of the diffusion speed, and the amplitude A_1 as the mobile fraction. We found that A_2 was generally small (about 5% of A_1) and τ_2 was very large (about $10\tau_1$). This term was more dependent on details of bleaching during the monitoring phase and did not reflect the true mobility behavior of the proteins.

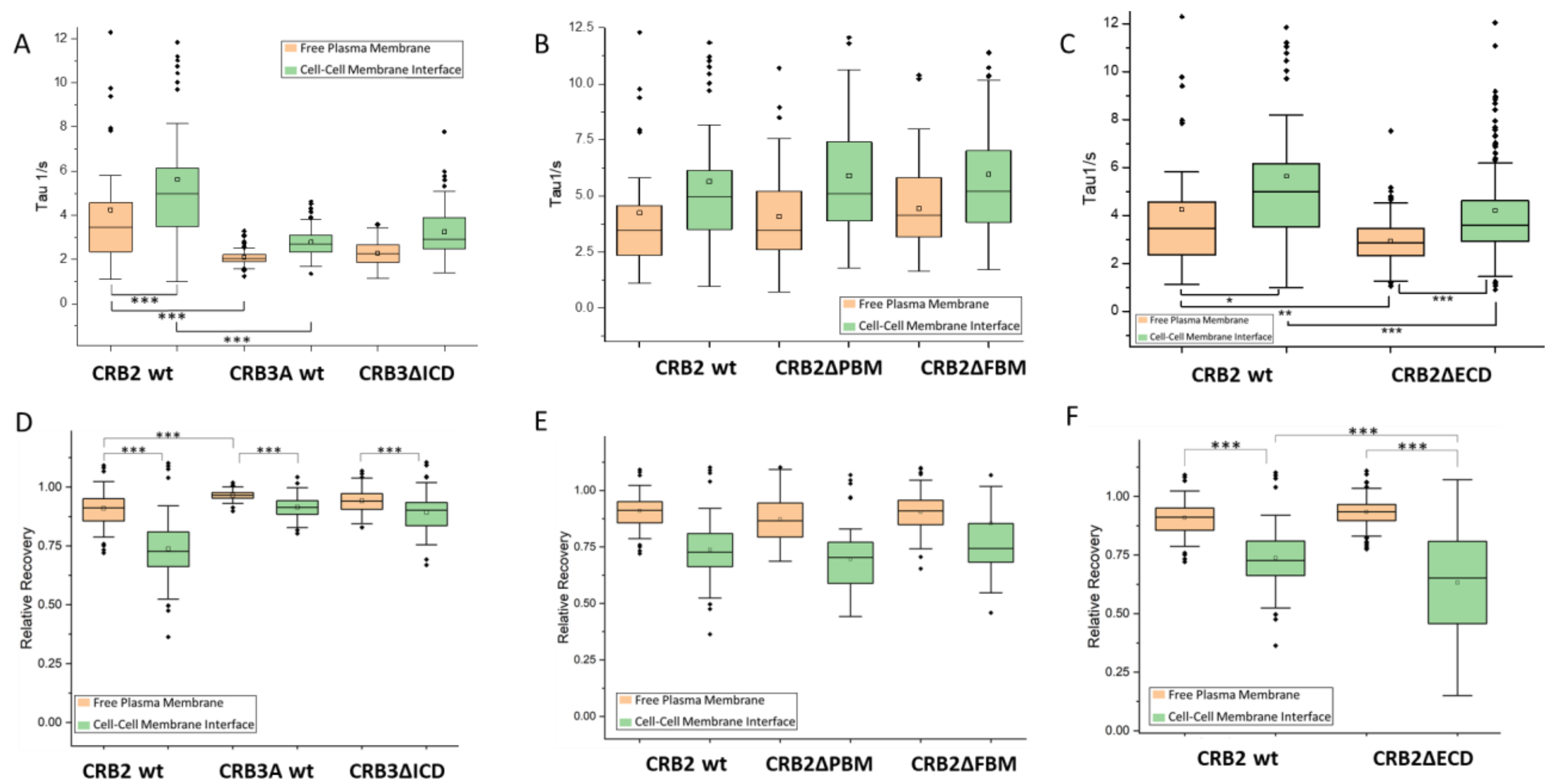

Fig. S4: Statistical analysis of FRAP measurements.

Each individual measurement was fitted to a bi-exponential curve as described. All single τ1 and maximum recovery values were plotted as box plots and tested for significance (*** - $p<0.001$, ** - $p<0.005$, * - $p<0.01$,). (A) CRB2 wt, CRB3A wt and CRB3ΔICD in free plasma membrane. (B) CRB2 wt, CRB2ΔPBM and CRB2ΔFBM in free plasma membrane regions. (C) CRB2 wt and CRB2ΔECD in free plasma membrane regions. (D) CRB2 wt, CRB3A wt and CRB3ΔICD at the cell-cell membrane interface. (E) CRB2 wt, CRB2ΔPBM and CRB2ΔFBM at the cell-cell membrane interface. (F) CRB2 wt and CRB2ΔECD at the cell-cell membrane interface.

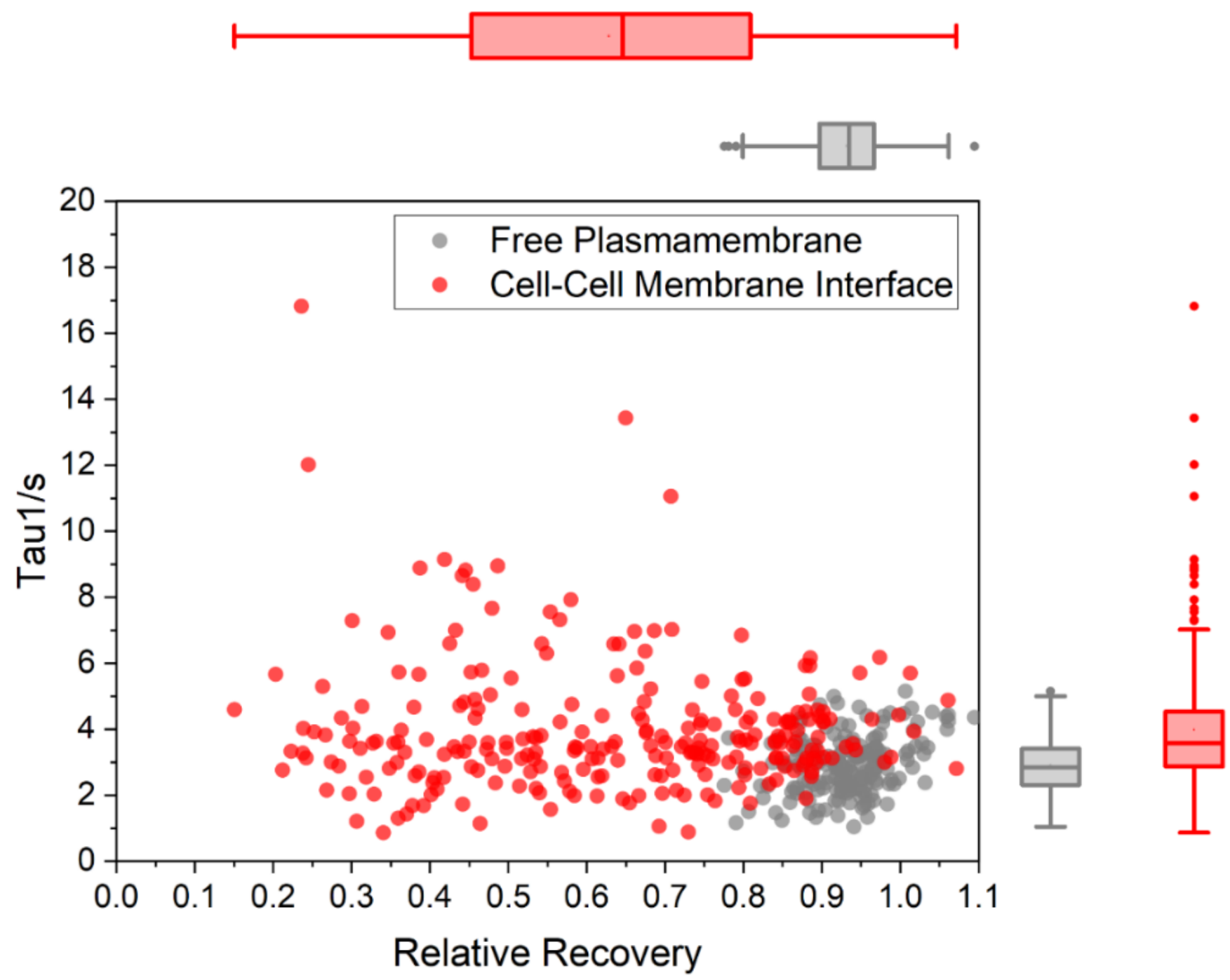

Fig. S5: Single FRAP Measurements of CRB2ΔECD in AB8/13 podocytes.

Combined scatter and box plot of all time constants τ_1 versus the respective maximum relative recovery values A_1 from single bi-exponential fits of all CRB2ΔECD FRAP measurements. For this mutant two complete triplicate sets of experiments comprising 30 individual measurements each corresponding to a total number of 180 single measurements were conducted.

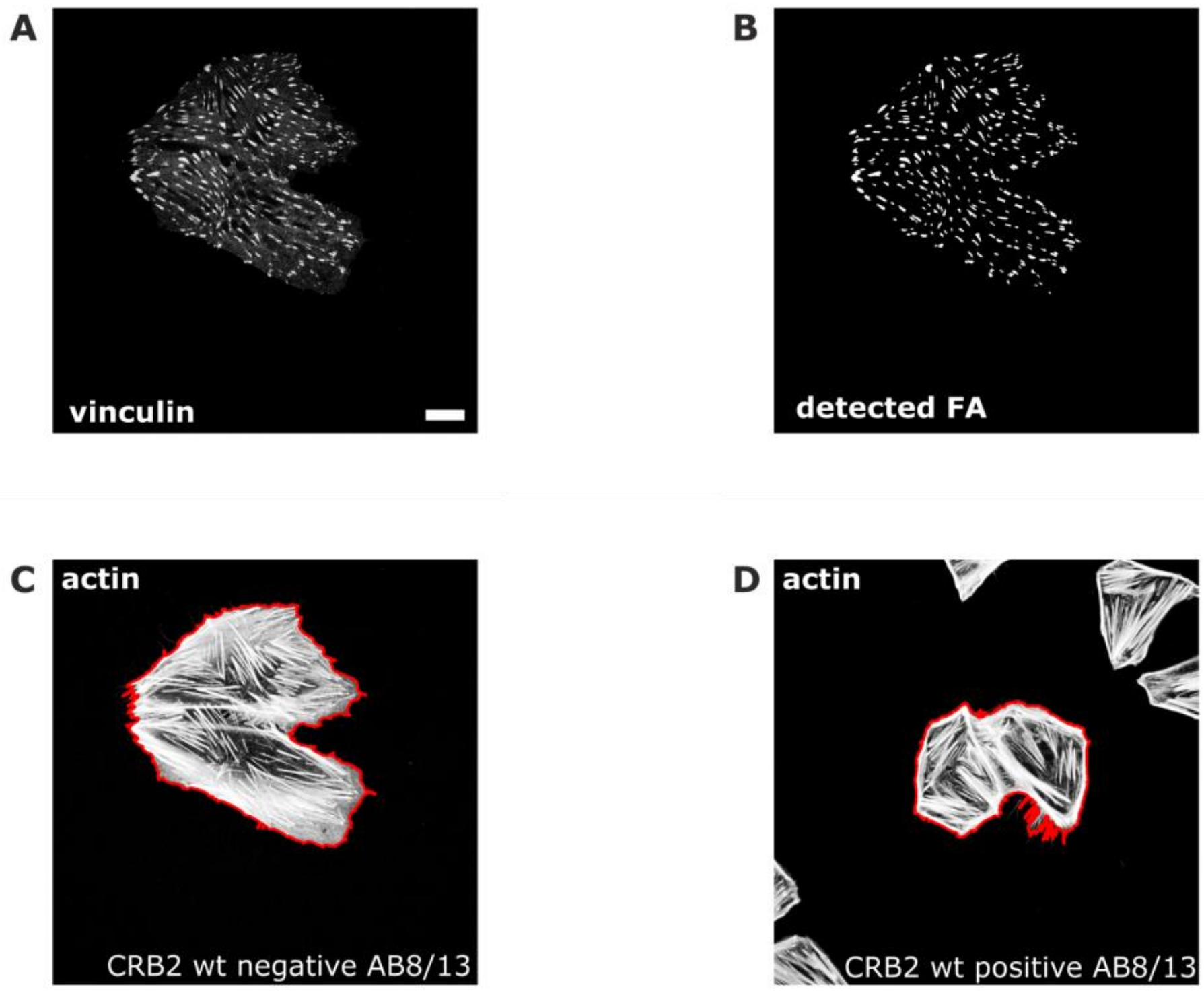

Fig. S6: Quantification of focal adhesions and actin distribution in AB8/13 cells.
(A) Immunofluorescent image of vinculin in AB8/13 cells. (B) Binary mask generated from (A) by z-scoring. (C) Automated detection of actin signal in CRB2 wt negative cell duplets. (D) Automated detection of actin signal in CRB2 wt positive cell duplets. In C and D the cell mask is indicated by a red line. Scale bar, 20 µm, applies to all.

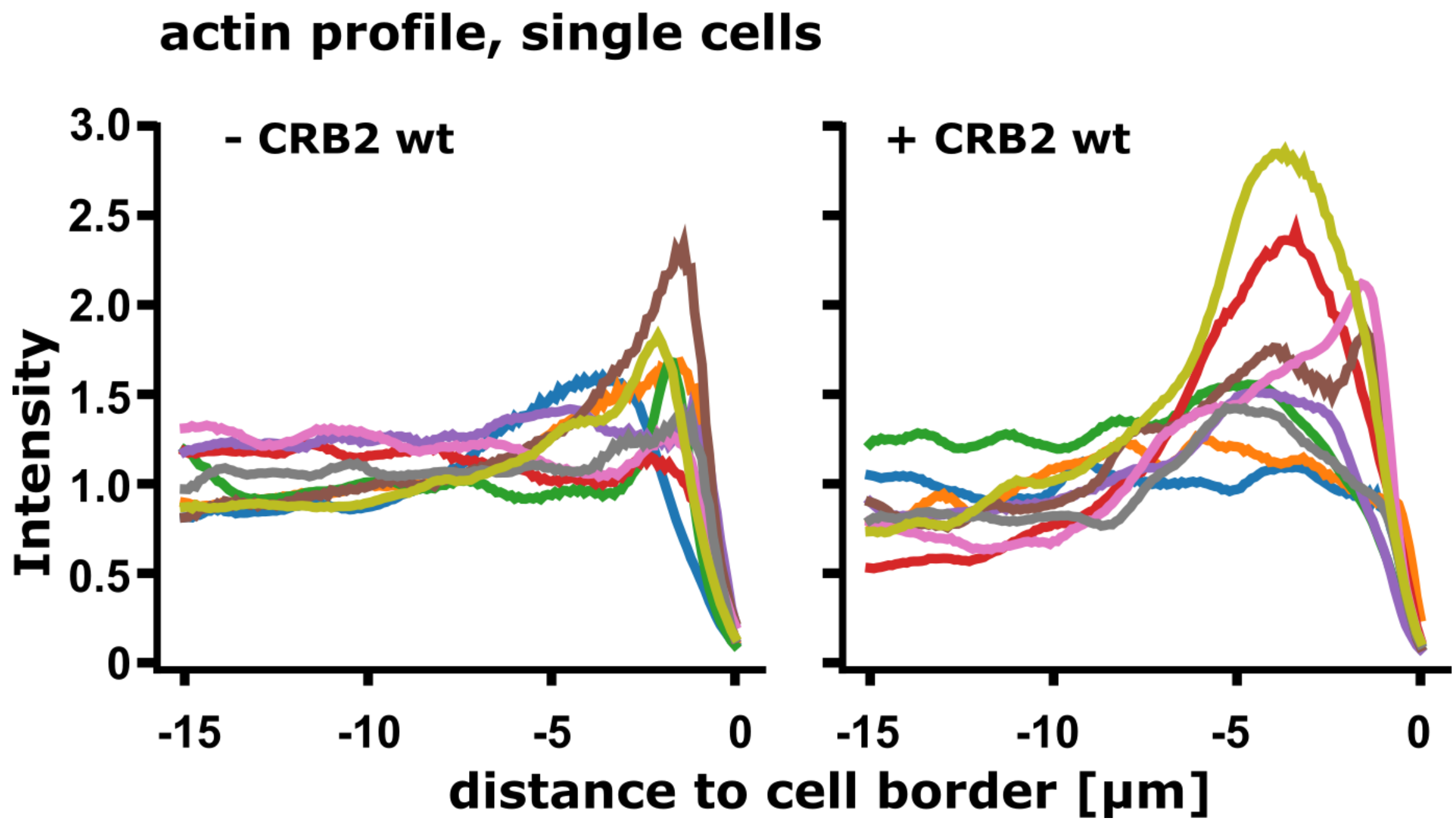

Fig. S7: Distribution of filamentous actin in nine isolated AB8/13 podocytes. Plotted are the mean grey value for each ring normalized to the mean grey value of the entire cell area. Left: CRB2-negative cells, right: CRB2-positive cells.

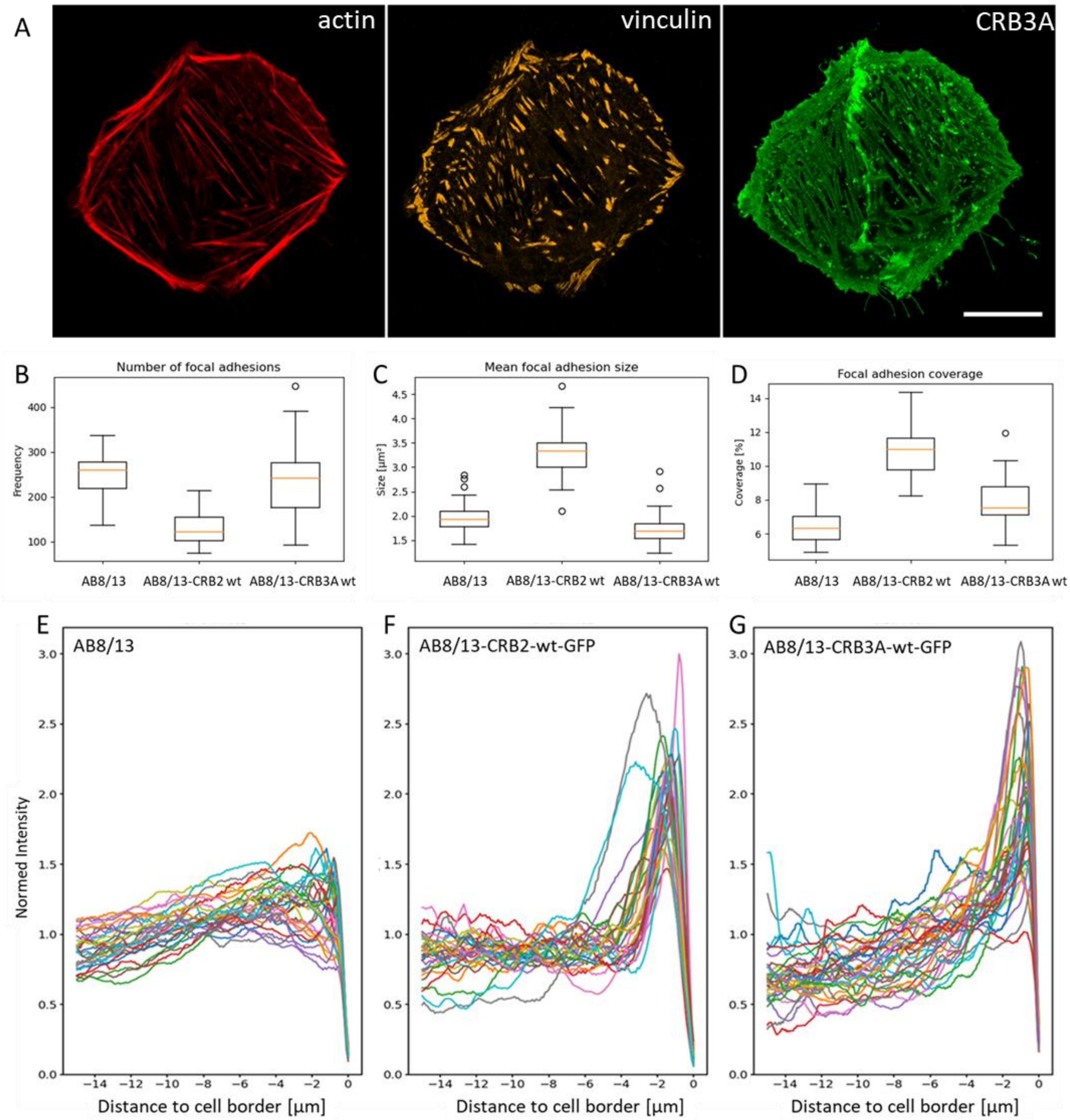

Fig. S8: Quantification of focal adhesions and actin distribution in CRB2-GFP and CRB3A-GFP AB8/13 cell duplets. (A) Immunofluorescence image of actin, vinculin and CRB3A-wt-GFP in AB8/13 cell duplets (scale bar, 20µm, applies to all). (B) Number of focal adhesion in AB8/13, AB8/13-CRB2-wt-GFP and AB8/13-CRB3A-wt-GFP. (C) Mean focal adhesion size in AB8/13, AB8/13-CRB2-wt-GFP and AB8/13-CRB3A-wt-GFP. (D) Focal adhesion coverage in AB8/13, AB8/13-CRB2-wt-GFP and AB8/13-CRB3A-wt-GFP. In B through D dots are results from individual duplets, horizontal bars depict ensemble quartiles and median. (E) Actin intensity profiles in AB8/13. (F) Actin intensity profiles in AB8/13-CRB2-wt-GFP. (G) Actin intensity profiles in AB8/13-CRB3A-wt-GFP.

Wound healing assay

The influence of CRB2 wt overexpression on cell migration was addressed using wound healing assays. Non-induced and doxycycline induced AB8/13 CRB2 wt cells were imaged over 24 h after applying a scratch through the confluent cell layer. Wound areas were recorded by light transmission microscopy at 0h and 24 h (Fig. S7A). Each condition was tested six times. Uninduced AB8/13 cells (-Dox) show a smaller open wound area than podocytes expressing CRB2 wt after 24 h (Fig. S7B).

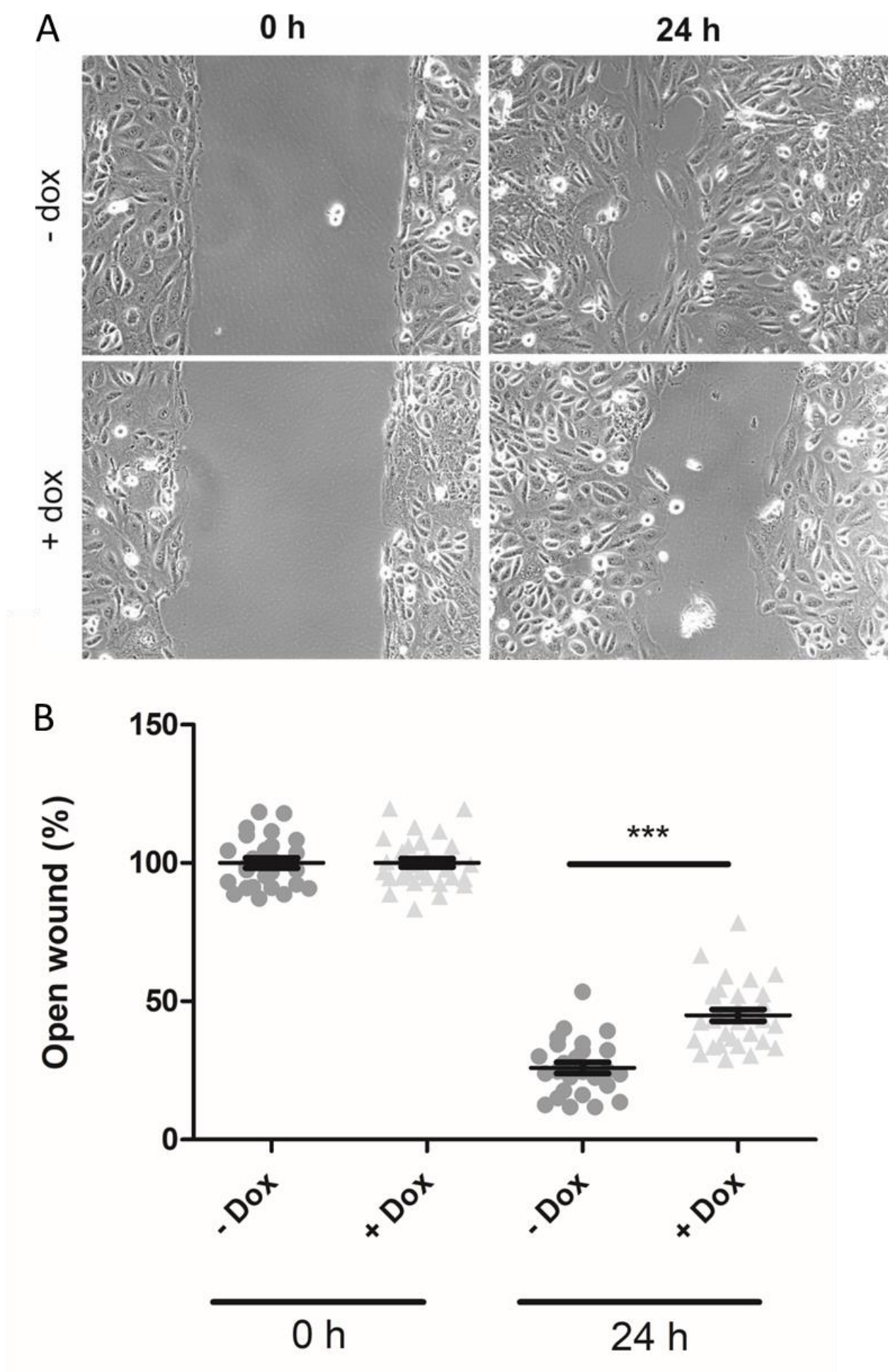

Fig. S9: Analysis of migratory behavior of CRB2 wt cells via wound healing assays.

(A) Migration of AB8/13 podocytes in which the expression of CRB2 wt was not induced (- dox) or was induced (+ dox) with doxycycline. The cell monolayer was imaged immediately after scratching and 24 hours later. (B) Percentage of cell coverage in the beginning and after 24 h of cell migration (*** $p \leq 0.001$).

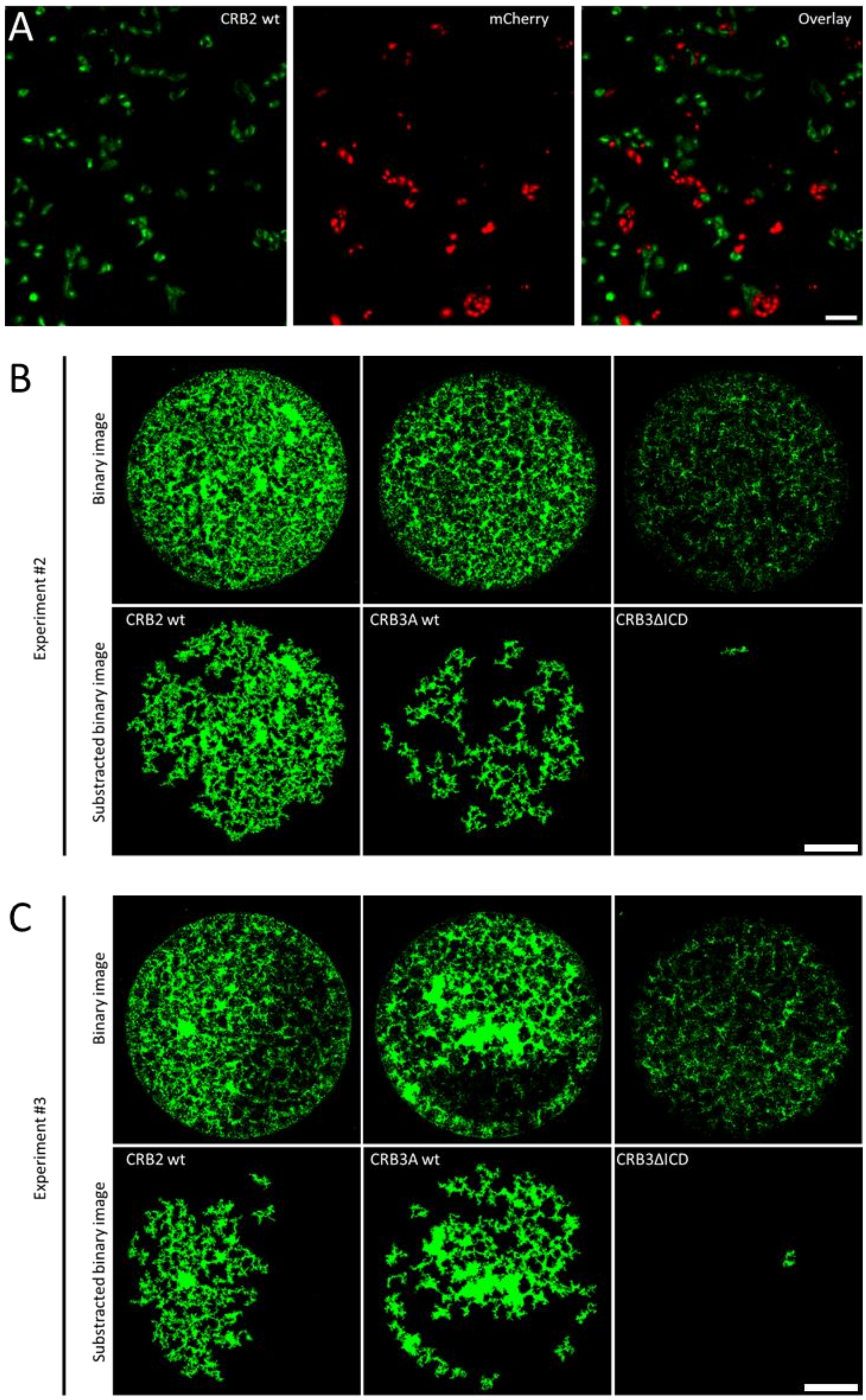

Fig. S10: Growth pattern analysis.

Co-cultivation of AB8/13 podocytes expressing either CRB2-wt-GFP or only mCherry. The complete cell culture dish was imaged using a confocal laser microscope (10x air objective, NA 0.45). (A) Cell distribution after one day of co-culture (size bar, 200 µm). (B) and (C) show the binary and subtracted binary images of two additional independent experiments after seven days (size bar, 5 mm).

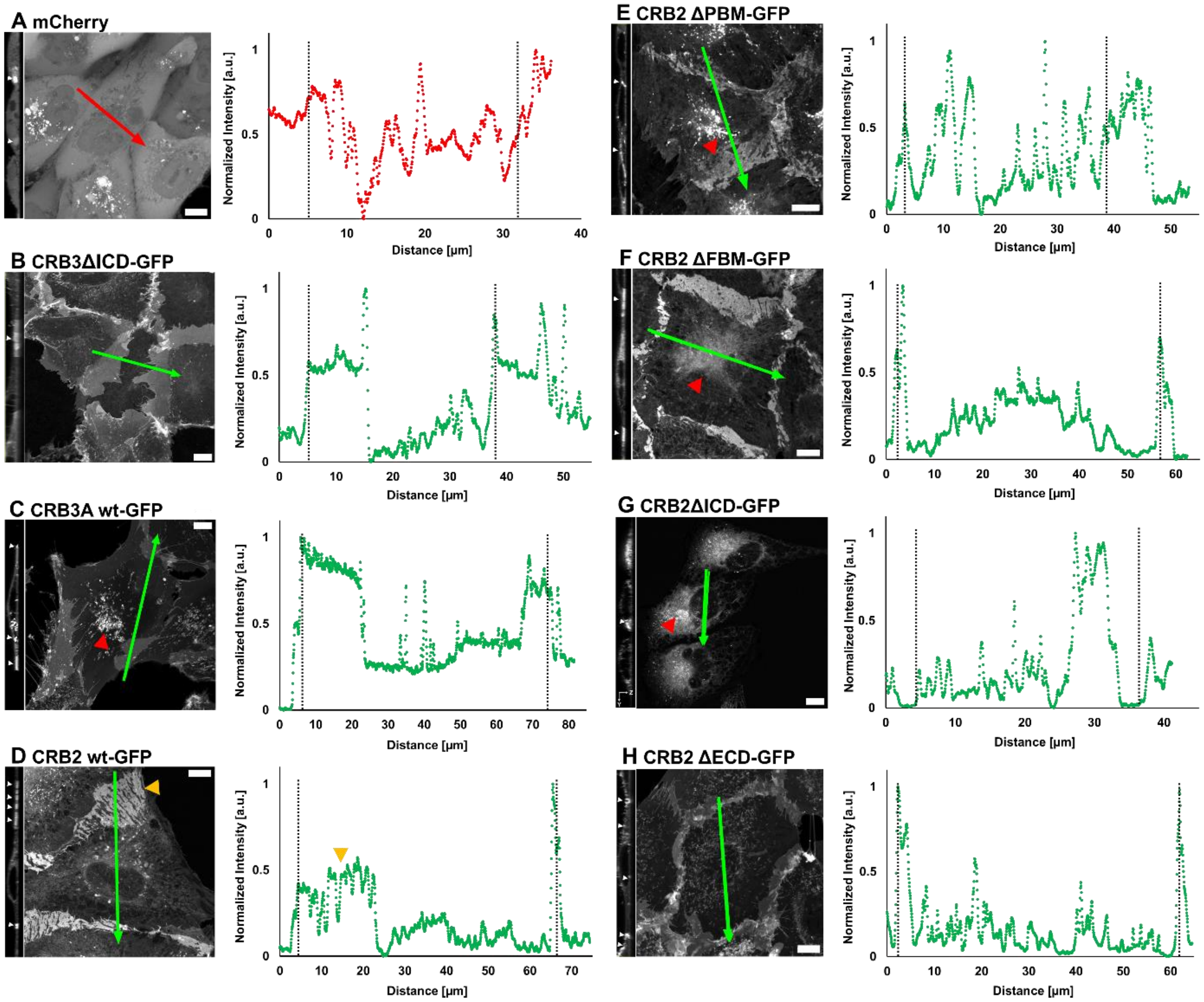

Fig. S11: Sub-cellular protein localization within CRBs isoforms.

(A) mCherry, (B) CRB3ΔICD-GFP, (C) CRB3A wt-GFP; (D) CRB2 wt-GFP, (E) CRB2 ΔPBM-GFP, (F) CRB2 ΔFBM-GFP, (G) CRB2ΔICD-GFP, and (H) CRB2ΔECD-GFP cell lines. Maximum intensity projections of representative Z-stacks (left) along with line profiles (right). Red arrowheads indicate ER localization, yellow arrowheads indicate a CRB2 wt specific morphology. Right figures: distance versus normalized intensity plot shown for the drawn line profile. Dotted lines represent cellular edges, where two cell membranes overlap. All images were acquired with a 40X water immersion objective; NA 1.2 on Zeiss LSM 880 with Airyscan detector. Scale bar: 10 µm.

It is obvious that the CRB2 mutants that we used in the FRAP experiments also show enrichment in the cell-cell membrane interfaces, but intriguingly only CRB2 wt shows a specific distribution within these cell-cell contact regions. Instead of a relative constant intensity we here see dark finger-like structures devoid of the CRB2 wt-GFP. From the immunostaining shown in Fig. 6 we can suppose that the dark regions correspond to focal adhesions according to the vinculin staining.
